# Do Epigenetic Clocks Provide Explanations for Sex Differences in Life Span? A Cross-Sectional Twin Study

**DOI:** 10.1093/gerona/glab337

**Published:** 2021-11-09

**Authors:** Anna Kankaanpää, Asko Tolvanen, Pirkko Saikkonen, Aino Heikkinen, Eija K Laakkonen, Jaakko Kaprio, Miina Ollikainen, Elina Sillanpää

**Affiliations:** Gerontology Research Center (GEREC), Faculty of Sport and Health Sciences, University of Jyväskylä, Jyväskylä, Finland; Methodology Center for Human Sciences, University of Jyväskylä, Jyväskylä, Finland; Gerontology Research Center (GEREC), Faculty of Sport and Health Sciences, University of Jyväskylä, Jyväskylä, Finland; Institute for Molecular Medicine Finland (FIMM), HiLife, University of Helsinki, Helsinki, Finland; Gerontology Research Center (GEREC), Faculty of Sport and Health Sciences, University of Jyväskylä, Jyväskylä, Finland; Institute for Molecular Medicine Finland (FIMM), HiLife, University of Helsinki, Helsinki, Finland; Institute for Molecular Medicine Finland (FIMM), HiLife, University of Helsinki, Helsinki, Finland; Department of Public Health, University of Helsinki, Helsinki, Finland; Gerontology Research Center (GEREC), Faculty of Sport and Health Sciences, University of Jyväskylä, Jyväskylä, Finland; Institute for Molecular Medicine Finland (FIMM), HiLife, University of Helsinki, Helsinki, Finland

**Keywords:** Biological age, DNA methylation, Life span, Lifestyle, Sex gap

## Abstract

**Background:**

The sex gap in life expectancy has been narrowing in Finland over the past 4–5 decades; however, on average, women still live longer than men. Epigenetic clocks are markers for biological aging which predict life span. In this study, we examined the mediating role of lifestyle factors on the association between sex and biological aging in younger and older adults.

**Methods:**

Our sample consists of younger and older twins (21‒42 years, *n* = 1 477; 50‒76 years, *n* = 763) including 151 complete younger opposite-sex twin pairs (21‒30 years). Blood-based DNA methylation was used to compute epigenetic age acceleration by 4 epigenetic clocks as a measure of biological aging. Path modeling was used to study whether the association between sex and biological aging is mediated through lifestyle-related factors, that is, education, body mass index, smoking, alcohol use, and physical activity.

**Results:**

In comparison to women, men were biologically older and, in general, they had unhealthier life habits. The effect of sex on biological aging was partly mediated by body mass index and, in older twins, by smoking. Sex was directly associated with biological aging and the association was stronger in older twins.

**Conclusions:**

Previously reported sex differences in life span are also evident in biological aging. Declining smoking prevalence among men is a plausible explanation for the narrowing of the difference in life expectancy between the sexes. Data generated by the epigenetic clocks may help in estimating the effects of lifestyle and environmental factors on aging and in predicting aging in future generations.

Both sexes have experienced tremendous increases in life expectancy over the twentieth century. However, through all historical periods, women have had a longer life expectancy than men. The sex gap in life expectancy varies across time and country (1). In Finland, the sex gap increased greatly in the first half of the twentieth century. That gap was greatest in the mid-1970s (9 years); since then, it has narrowed to 5.4 years (2).

It has been suggested that sex differences in life span are caused by a complex combination of biological (genetic, hormonal) and nonbiological (behavioral, economic, social, environmental, and cultural) factors (3). Investigating sex differences in cause-specific mortality increases the understanding of the mechanisms underlying the sex differences in overall mortality. In comparison to women, men experience a higher risk of death from almost all causes (4). External causes of deaths, such as traffic accidents, trauma, alcohol intoxication, illicit drug overdoses, and suicides, are more common among men. However, at most, these factors typically explain a modest fraction of all premature deaths. The majority of premature deaths are caused by noncommunicable diseases (eg, cardiometabolic diseases, lung diseases, cancers, mental disorders, and dementia) (4). The biological and behavioral factors predisposing an individual to these diseases are predominantly the most important drivers of male-to-female differences in mortality.

Overweight and obesity are dramatically increasing worldwide, predisposing both men and women to several noncommunicable diseases (5). While total body fat is lower in men, accumulation of harmful ectopic fat seems to be higher in men than women (6). Data regarding how obesity trends affect the sex gap in life expectancy are limited. Of the health-hazardous behavioral factors, tobacco smoking has been seen as the predominant driver of both the trend and the extent of sex differences in life expectancy. A recent study suggested that increasing smoking-related mortality among women and decreasing smoking-related mortality among men may account for as much as 40% of the narrowing sex gap in life expectancy over the last 2 decades (7). In general, men consume more alcohol than women. In Finland, the risk for alcohol-related death is 3 times higher in men than women (2). Globally, men tend to be more physically active than women at all ages (8), and leisure-time physical activity is known to be associated with a lower risk of premature death (9). Therefore, leisure-time physical activity is expected to diminish the sex gap in life expectancy. Socioeconomic factors might also affect the sex gap, such as differences in education, income, and physical demands of work between the sexes. For example, the sex gap is probably diminishing because many deaths related to trauma and toxication that previously occurred in male-dominated occupations are much rarer nowadays (5).

In addition to societal factors, differences in innate biology may also have a role in the survival gap between the sexes. Genetic and physiological differences between the sexes include progressive skewing of X chromosome inactivation, telomere attrition, maternally inherited mitochondrial inheritance, and hormonal and cellular differences in inflammatory and immunological responses and in substrate metabolism (10). The biological longevity advantage of women may also result from estrogen-associated greater resistance to oxidative damage (10). However, in women, sex hormone levels change drastically during menopause, potentially contributing to the reduction of age-related sex differences in health outcomes such as cardiovascular risk factors. The presentation of many diseases is directly influenced by biological factors as well as by gender identity associated with societal norms (3); through multiple routes, these are likely to contribute to the observed sex gaps in mortality and life expectancy.

Life expectancy may not always be a reliable proxy for how fast the population is aging, as it is the most distal outcome of aging processes. To better monitor population health, more sensitive methods are needed to track changes in aging. Novel biological clocks, that is, epigenetic clocks, may help track and understand the individual aging process and offer insights into sex differences in biological age and how lifestyle may counteract the aging process (11–15). These composite measures have been developed to quantify an individual’s biological age, and they may enable accurate estimation of the pace of aging in all age groups. The first published results on biological age determined by epigenetic clocks have shown that men tend to be biologically older than women (16–20).

This study aimed to examine sex differences in biological age measured by novel epigenetic clocks in age groups younger and older than 50 years, with 50 being a proxy for menopausal age (21). Moreover, we aimed to assess whether the potential difference in biological aging between the sexes is mediated by different lifestyle factors, and whether age modifies these associations.

## Method

### Study Population

The Finnish Twin Cohort (FTC) includes 3 large cohort studies: (a) The older FTC includes twins born before 1958, (b) Finntwin16 includes twins born in 1975–1979, and (c) Finntwin12 includes twins born in 1983–1987 (22–24). The older FTC was established 45 years ago, and data collection has been extensively described recently (24). Finntwin16 was initiated in 1991 and to date, it includes 5 waves of completed data collections (22). The main scope of the project is to identify the genetic and environmental determinants of various health-related behaviors and diseases in different stages of life. Finntwin12 is the youngest of the 3 FTC cohorts (23). All eligible twins born in Finland during 1983–1987 along with their biological parents were enrolled to participate in 4 waves of questionnaires. Selected twins took part in laboratory studies with repeated interviews, neuropsychological tests, and collection of DNA were made as part of Wave 4 in early adulthood (23).

Twins from all 3 cohorts (age range from 21 to 76 years) who had taken part in clinical in-person studies with sampling for whole-blood DNA and subsequent DNA methylation (DNAm) analyses and who had the relevant phenotype data were included in the current study. The analysis sample included monozygotic (MZ) and dizygotic (DZ) same-sex twins (*N* = 1 893, 54% MZ) as well as opposite-sex twins (347 twin individuals, 151 complete twin pairs). Zygosity of same-sex pairs was confirmed by multiple genetic markers from genome-wide array data.

The FTC data collections were approved by the ethics committees of the University of Helsinki (113/E3/01 and 346/E0/05) and Helsinki University Central Hospital (270/13/03/01/2008 and 154/13/03/00/2011).

### Main Variables

#### DNAm and assessment of biological age

DNAm profiles were obtained using Illumina’s Infinium HumanMethylation450 BeadChip or the Infinium MethylationEPIC BeadChip (Illumina, San Diego, CA). A more detailed description of the preprocessing and normalizing of the DNAm data is provided in Supplementary Material. We utilized 4 epigenetic clocks to produce biological age estimates. Horvath’s and Hannum’s versions incorporate methylation levels of 353 and 71 age-related CpGs, respectively, and were trained via regressing on chronological age through a penalized regression model (12,13). The third epigenetic age estimator, DNAm PhenoAge, was trained on a composite clinical measure of phenotypic age and includes 513 CpG sites (14). The newest epigenetic clock, DNAm GrimAge, includes 1 030 CpG sites and was a product of the 2-step development method (15). It first utilized DNAm data to predict a set of biomarkers (plasma proteins and smoking pack-year) and then these developed DNAm-based biomarkers were used to predict mortality. In both steps, information on participants’ sex and chronological age was used as well.

DNAm-based epigenetic age estimates, obtained by Horvath’s and Hannum’s clocks and by PhenoAge and GrimAge estimators, were calculated using a publicly available online calculator (https://dnamage.genetics.ucla.edu/new). The age acceleration (AA) of each clock was defined as the residual from regressing the estimated biological age on chronological age (AA_Horvath_, AA_Hannum_, AA_Pheno_, and AA_Grim_, respectively).

The components of DNAm GrimAge (adjusted for age) were obtained as well, including DNAm-based smoking pack-years and the surrogates for plasma proteins (DNAm-based plasma proteins): DNAm adrenomedullin (ADM), DNAm beta-2-microglobulin (B2M), DNAm cystatin C, DNAm growth differentiation factor 15 (GDF15), DNAm leptin, DNAm plasminogen activator inhibitor 1 (PAI-1), and DNAm tissue inhibitor metalloproteinases 1 (TIMP-1).

### Potential Mediating Variables

We surmised that differences in the covariates between the sex groups are more likely the factors that underlie the sex differences rather than being confounders. To study the factors underlying the sex differences, we chose lifestyle correlates that theoretically can be part of the mechanism leading to differences in cardiovascular diseases as well as in the length of the life span. The potential mediators included body mass index (BMI), smoking, alcohol use, physical activity, and educational attainment, which is a key component of socioeconomic status.

*Educational attainment* was assessed as the number of years of full-time education.

*BMI*, measured as kg/m^2^, can be used as an estimate of healthy diet and sufficient energy intake. A high BMI describes excess fat in the body; thus, it is a consequence of a long-term imbalance between energy intake and expenditure. We measured height in cm using a stadiometer and body mass in kg using a beam scale in kg.

*Smoking* was self-reported and classified as never, former, and current smokers.

*Alcohol use* was measured based on self-reported quantity and frequency of use and the content of the alcoholic beverages. These data were transformed into 100% alcohol grams per day.

*Physical activity* was assessed using the Baecke Questionnaire (25). The questionnaire has 3 sections: sports participation, leisure-time physical activity excluding sports, and work- or school-related physical activity. The questionnaire includes 4 questions on sports activity and leisure-time activity, excluding sports, and 8 questions on occupational physical load scored on a 5-point scale. A sport index, a nonsport leisure-time (leisure) index, and a work index, respectively, were based on the mean scores of each section as described by Baecke et al. (25) and Mustelin et al. for the FinnTwin12 study (26).

### Statistical Analysis

To compare differences in the study variables between men and women, we used linear regression analysis for the continuous variables and (multinomial) logistic regression for the categorical variables. In the models, the within-pair dependency of twin individuals was taken into account using the cluster option in the analysis.

Correlation coefficients between age and epigenetic age (DNAmAge) estimates and between AA measures were studied. The shape of the association between age and AA was studied using polynomial models of age as the continuous variable. To study whether sex differences in AA varied by age, the interaction effects of sex and age were also included in the regression models.

**Figure 1.**
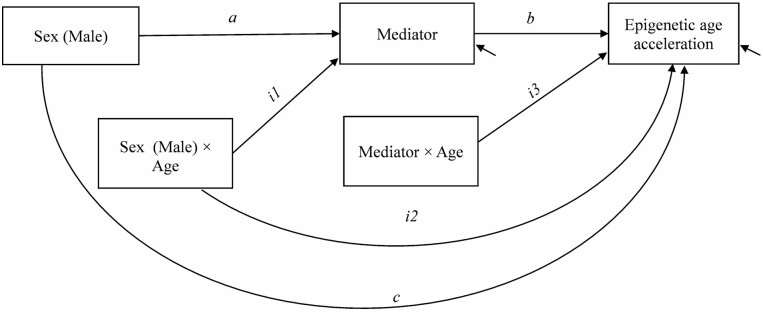
The path diagram of the single mediator model in all twins. The model included also the direct effect of age on the mediator and epigenetic age acceleration.

Mediation models were used to test whether the association between sex and AA is direct or mediated through lifestyle factors in all twins and opposite-sex twin pairs. First, the single mediation models were fitted. These models included indirect paths from sex to AA through one lifestyle factor at a time as well as the direct effect of sex on AA. In all twins, we further studied whether these associations differed according to age group, that is, whether age moderated the associations (Figure 1). The single mediation models included the interaction effect of sex and age group on the mediator variable (*i*1) and directly on AA (*i*2). Furthermore, the interaction effect between the mediator variable and age group on AA (*i*3) was tested for significance. Second, a multiple mediation model was fitted to assess the mediation effect of the different lifestyle factors simultaneously. The mediators as well as the interactions were included in the final multiple mediation model based on the results of the single mediator models.

The standard errors were corrected for nested sampling using the special option in Mplus (TYPE = COMPLEX). The models for the opposite-sex twin pairs were fitted using multilevel modeling, and the mediation models were specified at the within-twin pairs level. The approach controls for shared childhood environmental factors and partly for genetic factors.

The age-specific indirect effects of sex (male) on AA through a mediator variable were calculated using the parameters of the models and the following formula: (*a* + *i*1 *×* Age) *×* (*b + i*3 *×* Age) (27). The standardized indirect effects were reported as an effect size measure. These coefficients reflect the sex difference in AA explained by a certain mediator variable on a standardized scale. In the main analysis, age was treated as a dichotomous variable (0 = younger, 1 = older). As a sensitivity analysis, we reanalyzed the data using polynomial functions of age in the modeling to confirm that observed associations are not due to the differences in age distribution between sexes. For opposite-sex twin pairs, the indirect effects were calculated as the product of the within-twin pair level regression coefficients (*a × b*).

The parameters of the models were estimated using the full information maximum likelihood method with robust standard errors. For the models including the ordinal mediator variable smoking status, the estimation was conducted using a robust weighted least squares estimator. In that case, the mediator is assumed to be the continuous latent variable underlying ordinal smoking status. Descriptive statistics and differences in the study variables were calculated and tested using Stata 16 software (StataCorp LLC, College Station, TX), and further modeling was conducted with the Mplus statistical package (version 8.2) (28).

## Results

### Sex Differences in Lifestyle Factors

The characteristics of the younger and older twins and the opposite-sex twin pairs included in this study are presented in Table 1. In both younger and older groups, there were fewer men than women. Among the older twins, the men were younger and better educated than the women. The men belonging to an opposite-sex twin pair had a lower level of education in comparison to their twin sisters. The men had a higher BMI in young adulthood than the women. Among all twins, there were more current smokers among the men in comparison to the women; there was no sex difference in smoking among the opposite-sex twin pairs. In all the groups, the men consumed more alcohol than the women. Moreover, the men had a lower level of leisure index in all the groups in comparison to the women, but the men in the younger same-sex twin group had a higher level of sport index than women.

**Table 1.** Sex Differences in Lifestyle-Related Factors, DNA Methylation Age, and Age Acceleration (AA) Estimates According to Age Group in All Twins (*n* = 2 240) and in Opposite-Sex Twin Pairs (151 pairs)

	All Twins								Opposite-Sex Twin Pairs			
	21- to 42-Year-Old Twins (*N* = 1 477)				Over 50-Year-Old Twins (*N* = 763)				21- to 30-Year-Old Twin Pairs (*N* =151)			
	Women	Men	Sex Difference		Women	Men	Sex Difference		Women	Men	Sex Difference	
			Mean	p			Mean	p			Mean	p
*N*	792^a^	685^b^			621^c^	142^d^			151^e^	151^e^		
Zygosity, mz/dz	349/443	274/411			322/299	86/56						
Education, years	16.8 (3.6)	16.6 (3.6)	−0.2	.266	9.6 (3.3)	12.3 (4.1)	2.6	<.001	17.3 (0.3)	16.3 (0.3)	−1.0	.003
Age, years	24.4 (3.5)	24.8 (3.3)	0.3	.166	66.6 (4.7)	62.0 (3.8)	−4.5	<.001	23.9 (2.2)			
DNAmAge, est. years												
Horvath	31.1 (5.5)	32.5 (5.1)	1.3	<.001	65.5 (6.1)	64.2 (5.4)	−1.2	.057	31.1 (4.6)	31.7 (4.9)	0.6	.076
Hannum	19.7 (4.5)	20.9 (4.3)	1.2	.001	55.7 (5.9)	55.0 (5.4)	−0.7	.311	19.1 (3.4)	20.6 (4.0)	1.4	<.001
PhenoAge	15.5 (6.9)	14.7 (6.2)	−0.8	.057	55.7 (7.7)	56.7 (7.2)	1.0	.231	14.2 (5.4)	13.8 (5.8)	−0.4	.459
GrimAge	26.4 (4.5)	27.7 (4.5)	1.3	<.001	58.6 (5.0)	59.2 (5.8)	0.6	.348	25.8 (3.1)	27.1 (3.7)	1.3	<.001
DNAmAge acceleration												
AA_Horvath_	−0.4 (3.8)	0.6 (3.7)	1.1	<.001	−0.8 (3.6)	3.3 (5.2)	4.1	<.001	−0.1 (3.5)	0.6 (3.9)	0.6	.076
AA_Hannum_	−0.5 (3.4)	0.4 (3.2)	1.0	<.001	−0.7 (4.7)	2.6 (4.2)	3.2	<.001	−0.6 (3.0)	0.8 (3.6)	1.4	<.001
AA_Pheno_	0.3 (5.8)	−0.8 (5.1)	−1.1	<.001	−1.2 (7.2)	4.3 (6.0)	5.5	<.001	−0.7 (5.4)	−1.1 (5.7)	−0.4	.458
AA_Grim_	−0.5 (3.5)	0.5 (3.5)	1.0	<.001	−0.8 (3.6)	3.3 (5.2)	4.1	<.001	−0.7 (2.9)	0.6 (3.4)	1.3	<.001
*Lifestyle-related variables*												
BMI, kg/m²	23.4 (4.8)	24.3 (3.8)	0.9	.001	27.6 (4.9)	28.0 (4.5)	0.4	.408	22.5 (3.5)	23.9 (3.5)	1.4	<.001
Smoking, *n* (%)												
Never	406 (51.3)	300 (43.8)			480 (77.3)	63 (44.4)		<.001	66 (43.7)	61 (40.4)		.646
Former	155 (19.6)	141 (20.6)	1.2^f^	.214	90 (14.5)	53 (37.3)	4.5^f^	.002	50 (33.1)	48 (31.8)	1.0^f^	.882
Current	230 (29.1)	244 (35.6)	1.4^f^	.028	51 (8.2)	26 (18.3)	3.9^f^	.023	35 (23.2)	42 (27.8)	1.3^f^	.323
Alcohol, g/day	7.4(9.4)	15.2 (18.0)	7.9	<.001	4..1 (7.4)	11.3 (19.7)	7.2	<.001	7.8 (9.3)	16.4 (19.9)	8.5	<.001
Physical activity												
Work index	2.7 (0.7)	2.7 (0.7)	0.0	.757	2.3 (1.0)	2.5 (0.98)	0.2	.161	2.7 (0.7)	2.8 (0.8)	0.1	.379
Sport index	2.9 (0.8)	3.0 (0.8)	0.1	.013	3.1 (0.8)	3.0 (0.83)	−0.1	.386	3.0 (0.8)	2.9 (0.8)	0.0	.942
Leisure index	3.0 (0.6)	2.8 (0.6)	−0.2	.001	2.9 (0.6)	2.7 (0.61)	−0.2	.026	3.0 (0.6)	2.8 (0.6)	−0.3	.001

*Notes:* DNAmAge = DNA methylation age; BMI = body mass index. Values are means and standard deviations (BMI, alcohol, physical activity indexes) or numbers and percentages (smoking). Between sex difference (linear or multinomial logistic regression analysis adjusted with family relatedness) is significant when *p* < .050. In physical activity indices *N*: ^a^544‒556, ^b^440‒446, ^c^177‒184, ^d^139‒141, and ^e^91‒93. ^f^The odds ratio, never smokers were the reference category.

### Sex Differences in Epigenetic Aging

**Figure 2.**
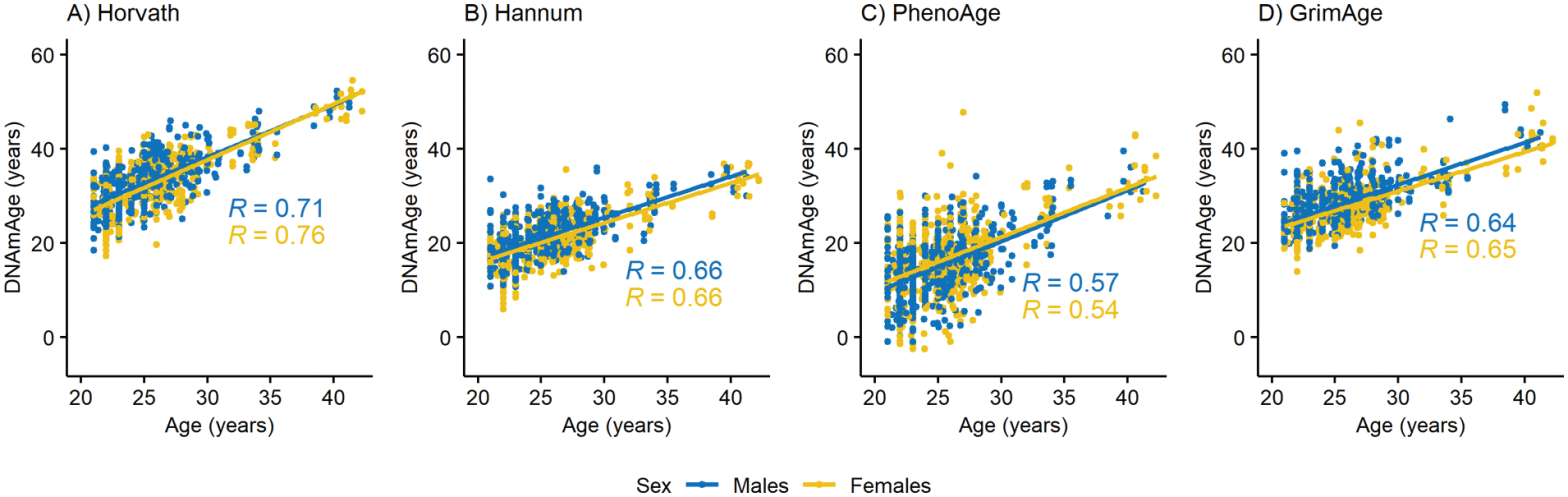
Association between chronological age and DNA methylation age (DNAmAge) estimates obtained by (A) Horvath’s clock, (B) Hannum’s clock, (C) PhenoAge, and (D) GrimAge estimators in younger (21- to 42-year-old) twins. *R* = Pearson’s correlation coefficient.

**Figure 3.**
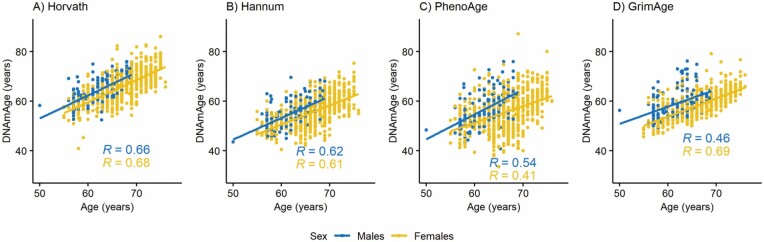
Association between chronological age and DNA methylation age (DNAmAge) estimates obtained by (A) Horvath’s clock, (B) Hannum’s clock, (C) PhenoAge, and (D) GrimAge estimators in older (older than 50 years old) twins. *R* = Pearson’s correlation coefficient.

The correlation coefficients between chronological age and DNAmAge estimates ranged from 0.54 to 0.76 in younger twins (Figure 2), from 0.41 to 0.69 in older twins (Figure 3), and from 0.23 to 0.69 in opposite-sex twins (Supplementary Figure 1). The correlation coefficients between AA measures ranged from 0.08 to 0.58 in younger twins, from 0.25 to 0.68 in older twins, and from −0.06 to 0.63 in opposite-sex twins (Supplementary Figure 2). The lowest correlation coefficients were observed between AA_Horvath_ and AA_Grim_, and the highest between AA_Hannum_ and AA_Pheno_.

Overall, the men had higher AA than the women, and the sex difference in AA tended to increase with age (Table 1 and Supplementary Table 1 and Supplementary Figure 3). Interestingly, when DNAm PhenoAge was used to assess AA, the men were epigenetically younger than the women in the younger age, which was in contrast with the AA estimates derived from the other clocks. When controlling for shared childhood environment and partly for genetic factors among the opposite-sex twin pairs, the male twins had higher AA_Hannum_ and AA_Grim_ in comparison to their sisters, but there were no significant differences in AA_Horvath_ and AA_Pheno_ between the sexes (Table 1).

### Mediation Models in All Twins

Education, BMI, smoking, alcohol use, sport index, leisure index, and work index were considered to be the potential mediator variables. Here, smoking was assumed to be a continuous latent variable underlying ordinal smoking status. The estimation results of the single mediator models revealed that the association of sex with education and smoking differed between the age groups (Figure 1, *i*1 and Supplementary Table 2). There were also some differences in the associations of the potential mediator variables (including education, smoking, and alcohol use) with AA between the age groups (*i*3). The results of the associations between lifestyle-related factors and AA are presented in Supplementary Material and Supplementary Table 2.

The estimated age-specific indirect associations of male sex on AA through the potential mediator variables are given in Table 2. Male sex was associated with higher AA_Pheno_ and AA_Grim_ through lower level of education in younger twins and with lower AA_Grim_ through higher level of education in older twins. Male sex was associated with higher AA_Horvath_ and AA_Pheno_ through higher BMI, but only in the younger twins. Smoking partly mediated the association of male sex with higher AA_Hannum_, AA_Pheno_, and AA_Grim_ in the older twins. Greater alcohol use partly mediated the association of male sex with higher AA_Pheno_ in the older twins and with AA_Grim_ in both age groups. Moreover, in younger twins, male sex was associated with lower AA_Grim_ through a higher sport index, but in older twins, with higher AA_Grim_ through lower leisure index. Work index did not mediate the sex difference in AA. Leisure index was chosen as an indicator of physical activity in further modeling.

**Table 2.** Standardized Indirect Effects of Sex (male) on Epigenetic Age Acceleration (AA) Through the Potential Mediator Variables in All Twins

	Mediator*^,^†^,^‡													
	Education		BMI		Smoking		Alcohol Use		Sport Index		Leisure Index		Work Index	
	*B* (*SE*)	*p*	*B* (*SE*)	*p*	*B* (*SE*)	*p*	*B* (*SE*)	*p*	*B* (*SE*)	*p*	*B* (*SE*)	*p*	*B* (*SE*)	*p*
*Single mediator models*														
Indirect associations in the younger twins														
AA_Horvath_	−0.007 (0.008)	.347	**0.014 (0.007)**	**.049**	−0.002 (0.013)	.895	−0.011 (0.012)	.372	0.005 (0.007)	.528	−0.003 (0.007)	.618	0.004 (0.009)	.699
AA_Hannum_	0.007 (0.007)	.358	0.002 (0.005)	.732	−0.001 (0.006)	.855	−0.011 (0.011)	.280	0.001 (0.007)	.899	−0.008 (0.008)	.321	−0.008 (0.011)	.435
AA_Pheno_	**0.020 (0.009)**	**.028**	**0.020 (0.009)**	**.020**	0.000 (0.001)	.965	−0.009 (0.011)	.386	−0.011 (0.009)	.227	−0.003 (0.008)	.700	−0.003 (0.009)	.787
AA_Grim_	**0.053 (0.016)**	**.001**	0.013 (0.007)	.057	0.000 (0.030)	.902	**0.045 (0.008)**	**<.001**	−**0.023 (0.011)**	**.044**	0.006 (0.007)	.358	−0.029 (0.023)	.199
Indirect associations in the older twins														
AA_Horvath_	−0.001 (0.009)	.947	0.005 (0.004)	.182	0.013 (0.010)	.170	−0.005 (0.010)	.589	0.000 (0.001)	.852	0.004 (0.006)	.534	0.01 (0.013)	.425
AA_Hannum_	0.008 (0.010)	.417	0.003 (0.002)	.224	**0.042 (0.013)**	**.001**	0.014 (0.009)	.109	0.000 (0.001)	.910	0.000 (0.007)	.980	−0.007 (0.011)	.495
AA_Pheno_	0.006 (0.010)	.517	0.006 (0.004)	.182	**0.063 (0.017)**	**<.001**	**0.037 (0.01)**	**<.001**	0.001 (0.002)	.734	−0.003 (0.008)	.655	−0.002 (0.01)	.881
AA_Grim_	−**0.025 (0.011)**	**.020**	0.006 (0.004)	.167	**0.160 (0.040)**	**<.001**	**0.048 (0.01)**	**<.001**	0.004 (0.008)	.609	**0.018 (0.009)**	**.042**	−0.005 (0.012)	.677
*Multiple mediator models* §														
Indirect associations in the younger twins														
AA_Horvath_	0.000 (0.005)	.958	**0.008 (0.004)**	**.030**	−0.002 (0.014)	.874	−0.003 (0.007)	.663	—‖		0.002 (0.005)	.672	—‖	
AA_Hannum_	0.001 (0.005)	.787	0.003 (0.003)	.328	−0.002 (0.007)	.825	0.001 (0.006)	.895			−0.004 (0.005)	.421		
AA_Pheno_	0.017 (0.017)	.322	**0.010(0.005)**	**.029**	0.000 (0.002)	.885	−0.007 (0.010)	.501			−0.006 (0.005)	.164		
AA_Grim_	0.005 (0.010)	.610	**0.006 (0.003)**	**.037**	0.004 (0.024)	.866	0.006 (0.005)	.181			0.004 (0.004)	.247		
Indirect associations in the older twins														
AA_Horvath_	0.000 (0.007)	.958	**0.008 (0.004)**	**.030**	0.016 (0.010)	.131	−0.003 (0.007)	.663	—‖		0.002 (0.005)	.672	—‖	
AA_Hannum_	0.001 (0.005)	.787	0.003 (0.002)	.139	**0.044 (0.013)**	**.001**	0.001 (0.006)	.895			−0.004 (0.005)	.421		
AA_Pheno_	0.012 (0.015)	.403	**0.010 (0.005)**	**.029**	**0.061 (0.017)**	**<.001**	**0.024 (0.01)**	**.013**			−0.006 (0.005)	.164		
AA_Grim_	−**0.029 (0.015)**	**.046**	**0.006 (0.003)**	**.037**	**0.161 (0.038)**	**<.001**	0.006 (0.005)	.181			0.004 (0.004)	.247		

*Notes:* BMI = body mass index; *B* = standardized (STDYX) indirect effect; *SE* = standard error.

*The model was controlled for zygosity.

^†^ *SE*s were corrected for nested sampling.

^‡^Significant regression coefficients at the level 0.05 are presented in bold.

^§^The indirect effects were estimated as equal in the younger and older twins whenever interaction terms were not needed.

^‖^Based on the results of the single mediator models, the corresponding mediator variable was dropped out from the final multiple mediator model.

**Figure 4.**
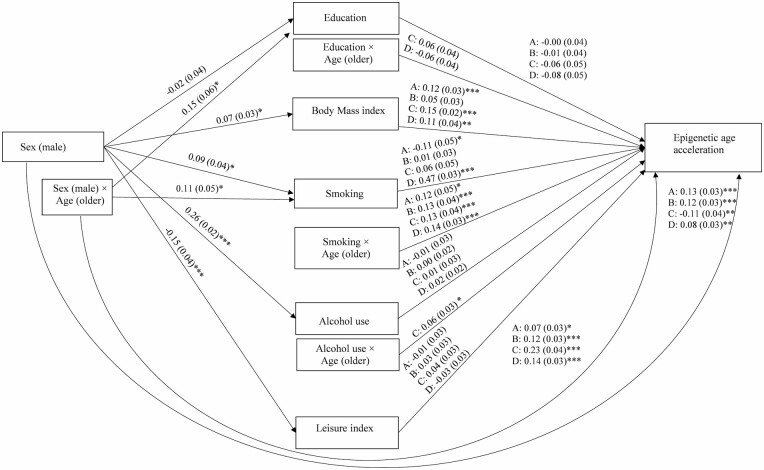
The path diagram of the multiple mediator model in all twins (*n* = 2 240). Standardized regression coefficients (standard errors) are presented. The modeling was conducted separately for each epigenetic age acceleration (AA) measure: (A) AA_Horvath_, (B) AA_Hannum_, (C) AA_Pheno_, and (D) AA_Grim_. ***p < .001, **p < .01, *p < .05.

Based on the results of the single mediator models, the final multiple mediator model included education, BMI, smoking, alcohol use, and leisure index as the mediators (Figure 4). The model also included the interaction effect between sex and age on education, the interaction effect between sex and age on smoking, and the interaction effect between smoking and age on AA. Furthermore, the interaction effect between education and age on AA_Pheno_ and AA_Grim_ and the interaction effect between alcohol use and age on AA_Pheno_ were included in the model. When the lifestyle factors were controlled for each other, male sex was associated with lower AA_Grim_ through higher level of education in older twins (Table 2). BMI partly mediated the association between sex and AA (AA_Horvath_, AA_Pheno_, and AA_Grim_). Moreover, smoking partly mediated the association between sex and AA (AA_Hannum_, AA_Pheno_, and AA_Grim_) but only in the older twins. Alcohol use partly mediated the sex difference in AA_Pheno_ in the older twins. Male sex was still found to have a direct positive effect on AA_Horvath_, AA_Hannum_, and AA_Grim_ after all the adjustments, and the association was stronger in the older cohort (Figure 4). Moreover, male sex was found to have a positive direct effect on AA_Pheno_, but only in the older cohort.

### Sensitivity Analysis

Finally, we reanalyzed the data using polynomial functions of age. The results were very similar to the ones obtained in the main analysis, but there were few exceptions (Supplementary Material, Supplementary Tables 3 and 4, and Supplementary Figures 4‒17). Importantly, based on the multiple mediator models, the indirect effects of male sex on AA through BMI, smoking, and alcohol use were consistent with ones observed in the main analysis.

### Mediation Models in the Opposite-Sex Twin Pairs

**Figure 5.**
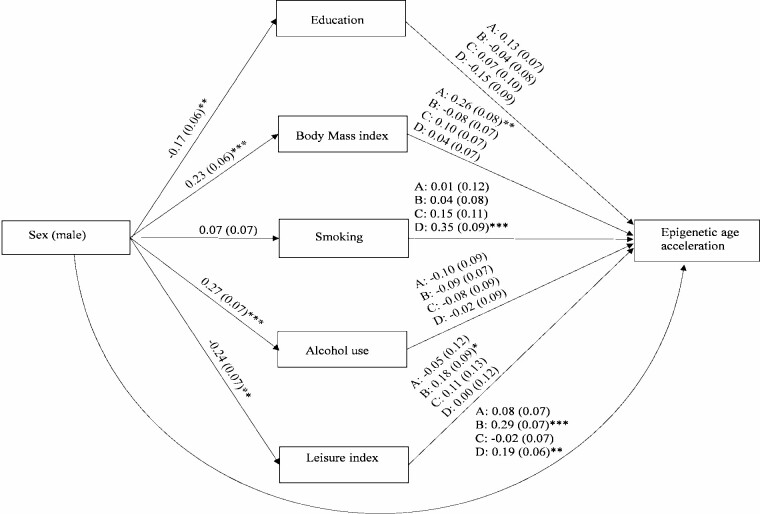
The path diagram of the multiple mediator model in the opposite-sex twin pairs (151 twin pairs). The standardized regression coefficients (standard errors) at the within-twin pair level are presented. The modeling was conducted separately for each epigenetic age acceleration (AA) measure: (A) AA_Horvath_, (B) AA_Hannum_, (C) AA_Pheno_, and (D) AA_Grim_. ***p < .001, **p < .01, *p < .05.

Information on the association between the lifestyle-related factors and AA is provided in Supplementary Text and Supplementary Table 5. Based on the estimation results of the single mediator models, male sex was associated with accelerated AA_Horvath_ through higher BMI in the opposite-sex twin pairs (Table 3). Otherwise, there were no significant indirect effects. Similar to the models for all twins, education, BMI, smoking, alcohol use, and leisure index were included in the multiple mediator model as the mediator variables (Figure 5). A significant indirect association of male sex on AA_Horvath_ through higher BMI was also observed after controlling for other lifestyle factors (Table 3). Otherwise, lifestyle factors did not mediate the differences in AA between the men and their female twin sisters. A direct effect of male sex on higher AA_Hannum_ and AA_Grim_ was also observed among the opposite-sex twin pairs (Figure 5).

**Table 3.** Standardized Indirect Effects of Sex (male) on Epigenetic Age Acceleration (AA) Through the Potential Mediator Variables in the Opposite-Sex Twins at Within-Twin Pair Level

	Mediator*^,^†													
	Education		BMI		Smoking		Alcohol Use		Sport Index		Leisure Index		Work Index	
	*B* (*SE*)	*p*	*B* (*SE*)	*p*	*B* (*SE*)	*p*	*B* (*SE*)	*p*	*B* (*SE*)	*p*	*B* (*SE*)	*p*	*B* (*SE*)	*p*
*Single mediator models*														
AA_Horvath_	−0.019 (0.016)	.251	**0.054 (0.022)**	**.015**	−0.003 (0.009)	.770	−0.026 (0.021)	.200	0.002 (0.002)	.847	−0.001 (0.018)	.957	−0.009 (0.010)	.385
AA_Hannum_	−0.010 (0.014)	.480	−0.012 (0.023)	.605	−0.004 (0.008)	.628	−0.032 (0.022)	.159	0.000 (0.010)	.985	−0.026 (0.020)	.186	0.001 (0.005)	.893
AA_Pheno_	−0.017 (0.012)	.173	0.015 (0.020)	.458	0.008 (0.011)	.460	−0.018 (0.020)	.366	0.001 (0.011)	.959	−0.025 (0.016)	.131	0.003 (0.006)	.640
AA_Grim_	0.025 (0.015)	.095	−0.006 (0.024)	.804	0.029 (0.029)	.307	0.009 (0.023)	.689	0.000 (0.001)	.939	0.022 (0.018)	.221	0.006 (0.008)	.411
*Multiple mediator models*														
AA_Horvath_	−0.022 (0.014)	.116	0.060 (0.025)	.019	0 (0.009)	.955	−0.028 (0.025)	.263	—‡		0.013 (0.028)	.646	—‡	
AA_Hannum_	0.006 (0.013)	.665	−0.018 (0.016)	.255	0.003 (0.006)	.677	−0.023 (0.019)	.222			−0.042 (0.025)	.096		
AA_Pheno_	−0.019 (0.028)	.503	0.035 (0.027)	.192	0.018 (0.022)	.394	−0.034 (0.041)	.403			−0.040 (0.05)	.416		
AA_Grim_	0.023 (0.016)	.161	0.007 (0.013)	.589	0.023 (0.023)	.306	−0.005 (0.021)	.816			0.001 (0.025)	.977		

*Notes:* BMI = body mass index; *B* = standardized (STDYX) indirect effect; *SE* = standard error.

*The model was controlled for age as twin pairs participated in measurements at slightly different ages (±1 year).

^†^Significant indirect effects at the level 0.05 are presented in bold.

^‡^Based on the results of the single mediator models in the same-sex and the opposite-sex twin pairs, the corresponding mediator variable was dropped out from the final multiple mediator model.

### Sex Differences in the DNAm-Based Plasma Proteins and Smoking Pack-Years

Information on sex differences in DNAm-based surrogates included in the DNAm GrimAge estimator is given in Supplementary Text, Supplementary Table 6, and Supplementary Figures 18 and 19.

## Discussion

Our findings suggest that previously reported sex differences in life expectancy can be seen in biological aging when measured with epigenetic clocks (namely Horvath’s clock, Hannum’s clock, DNAm PhenoAge, and GrimAge). Sex difference was already evident in young adulthood, and it increased with age; on average, the men were 1.2–1.3 years older than women in the younger twins (21–42 years of age) and 3.2–4.3 years older in the older twins (50–76 years of age). The only exception was observed in the younger twins when PhenoAge was used to assess biological aging; the men were epigenetically 1.1 years younger than the women, but the sex difference in biological age reversed in the older adult twins. According to previous studies, sex difference in biological aging measured with epigenetic clocks seems to appear in adolescence (29), and men are epigenetically 1–2 years older than women in adulthood (30).

Opposite-sex twins provide a natural setting for studying sex differences while maximally controlling for genetic factors and shared childhood environmental factors. Epigenetic aging is highly heritable (31). Although the share of genes is 50% in male–female dizygotic twins, the mean difference by sex of epigenetic aging within these twin pairs (21–30 years of age) was comparable to the sex differences observed in the larger cohort of younger twins.

The observed increase in sex difference with age was mainly due to the fact that epigenetic aging accelerated with age among men. Changes in the hormonal levels during menopause have a detrimental effect on women’s health (32); thus, sex differences in biological aging would be expected to diminish around and after the age of 50. Our analysis studying the shape of the association between chronological age and epigenetic aging did not find any evidence that this is the case in biological aging (Supplementary Material, Supplementary Table 1, and Supplementary Figure 3). This is in line with a recently published study investigating sex differences in the longitudinal trajectories of epigenetic aging from midlife onward (50–90 years) (18). According to the study, men were biologically older than women when Horvath’s and Hannum’s epigenetic clocks and GrimAge were used, and the difference remained constant across the age span (18).

To the best of our knowledge, this is the first study that has tested the mechanisms underlying sex differences in biological aging measured with epigenetic clocks. We found that several lifestyle-related factors partly mediated the association of sex with biological aging in all twins when the mediation of these factors was assessed one at a time. However, after controlling for each of the health-related behaviors in the multiple mediator models, BMI consistently mediated the sex difference in all twins and smoking in the older twins. Smoking was associated with accelerated biological aging, and the association was stronger in the older twins; this suggests the cumulative effect of smoking on biological aging. Moreover, the sex difference in smoking behavior was larger in the older twins; in fact, the difference in the proportion of never smokers between men and women was wider in the older twins (45% vs 73%) in comparison to the younger twins (44% vs 51%). A previous population-based study investigating long-term trends in smoking in Finland has shown that the prevalence of daily smoking has steadily decreased among men since the late 1970s (37%–17%); in contrast, the current prevalence among women is about the same as it was 4 decades ago (~15%) (33). Together, these findings support recent studies suggesting that the narrowing of the sex differences in smoking probably partly explains the declining sex gap in life span (7,34).

We observed some differences in the associations between lifestyle-related factors and epigenetic aging across the utilized clocks. These inconsistencies are probably due to differences in the procedures used to develop these epigenetic age estimators. The first-generation clocks, namely Horvath’s clock and Hannum’s clock, were trained to predict chronological age. More novel estimators are supposed to also capture CpG sites whose DNAm levels correlate with the deviation of biological age from chronological age.

Of the epigenetic age estimators employed in our study, DNAm GrimAge is the one that has been most recently published, and it outperforms other estimators in terms of predicting mortality (15,18). It is a mortality predictor by design and therefore may be the most relevant epigenetic age estimator in understanding sex differences in life expectancy. DNAm GrimAge utilizes information on chronological age, sex, and 7 DNAm-based surrogates for 7 plasma proteins and for smoking pack-years. Although sex difference in GrimAge is in-built, reflecting differences in mortality, the observed sex differences were very similar to the corresponding ones measured with Hannum’s clock, which is purely based on CpG sites with their DNAm levels correlating with chronological age. To further understand the sex differences in biological aging, we studied the DNAm-based surrogates included in the GrimAge estimator (Supplementary Material, Supplementary Table 6, and Supplementary Figures 18 and 19). We observed a significantly higher level of DNAm-based PAI-1 among men in comparison to women. Moreover, in men, the level of DNAm PAI-1 drastically increased with age. This DNAm-based surrogate predicts morbidity better than DNAm GrimAge, and it associates with hypertension, type 2 diabetes, and coronary heart disease (15). Therefore, higher levels of DNAm PAI-1 in men may play a role in the sex differences in cardiovascular mortality observed in previous studies (4).

Our study has several strengths. We utilized recently published epigenetic clocks that are shown to predict mortality (18,35). The large sample size of our study enabled us to use complex mediator models. Because data from opposite-sex twin pairs were available, we were also able to control the analyses for shared childhood environmental factors and partly for genetic factors. This study also had some limitations. Most of the studied lifestyle-related factors were self-reported. Furthermore, our data were cross-sectional, and our analysis did not rule out the possibility of reversed causality when studying the associations between lifestyle-related factors and epigenetic aging.

Our results deepen the understanding of the association between sex-dependent lifestyle factors and the aging process. The results suggest that sex difference in life span is narrowing among future aging generations, and the main reason for this is that at the mean level women and men are approaching each other in life habits, especially in smoking, which is rapidly declining in men. Progress in the methodology of biological aging measurements may enable us to determine individual trajectories in aging already in early adulthood. This makes it possible to investigate the effects of environmental and societal changes and lifestyle interventions on biological aging. Produced knowledge would help in preparing our societies for the aging of future generations.

## Supplementary Material

glab337_suppl_Supplementary_Material
