# Supplementary material for: Do Epigenetic Clocks Provide Explanations for Sex Differences in Life Span? A Cross-Sectional Twin Study
Source: J Gerontol A Biol Sci Med Sci. 2021 Nov 9;77(9):1898–906. doi: 10.1093/gerona/glab337 (PMC9434475; doi:10.1093/gerona/glab337)
Supplement: glab337_suppl_Supplementary_Material [file glab337_suppl_supplementary_material.pdf]

Supplementary Text

DNA methylation analysis

Genomic DNA was extracted from peripheral blood samples using commercial kits. High molecular weight DNA samples (1 µg) were bisulfite converted using EZ-96 DNA methylation-Gold Kit (Zymo Research, Irvine, CA, USA) according to the manufacturer's protocol. The twins and co-twins were randomly distributed across plates, with both twins from a pair on the same plate. DNA methylation (DNAm) profiles were obtained using Illumina's Infinium HumanMethylation450 BeadChip or the Infinium MethylationEPIC BeadChip (Illumina, San Diego, CA, USA). The Illumina BeadChips measure single-CpG resolution DNAm levels across the human genome. With these assays, it is possible to interrogate over 450,000 (450k) or 850,000 (EPIC) methylation sites quantitatively across the genome at single-nucleotide resolution. Methylation data were preprocessed using R package *minfi*. Detection *p* values comparing total signal for each probe to the background signal level, were calculated to evaluate quality of the samples (1). Samples of poor quality (mean detection $p > .01$) were excluded from further analysis. Data were normalized by using the single-sample Noob normalization method, which is suitable for datasets originating from different platforms (2). Beta values representing CpG methylation levels were calculated as ratio of methylated intensities (M) to the overall intensities (Beta value=M/(M+U+100), where U is unmethylated probe intensity). β values of the selected probes were used as the input to calculate the predicted age by different epigenetic age estimators using publicly available online calculator (https://dnamage.genetics.ucla.edu/new).

Results

Sex differences in epigenetic aging

Among all twins, the shape of the association between age and AA was studied using polynomial (cubic, quadratic and linear) models of age (Supplementary Table S1). The polynomial with the highest order term and significant regression coefficients was considered optimal. To study whether sex differences in age acceleration (AA) varied by age, the interaction effects of sex and age (Sex × Age^3, Sex × Age^2 and Sex × Age) were also included in the regression models. Higher-order interactions (Sex × Age^3, Sex × Age^2) were freed only when necessary (modification index > 4). For $AA_{Horvath}$ a third order term (Age^3) in cubic model was significant suggesting that the association between age and $AA_{Horvath}$ followed a cubic curve. There was a difference in the second order terms between the sexes (Sex × Age^2). The men were biologically older than the women at younger age, but the difference disappeared after 30 years of age and widened again after 50 years of age (Supplementary Figure S3, A). For AA_{Pheno} a second order term was significant and therefore, the quadratic model was chosen (Supplementary Table S1). There was a difference in both a linear term and quadratic term between sexes (Sex × Age and Sex × Age^2). Men had lower level of AA_{Pheno} at younger age, but the difference was opposite and increased steeply after 50 years of age (Supplementary Figure S3, C). The linear model was sufficient for AA_{Hannum} and AA_{Grim} (Supplementary Table S1). The men had higher level of AA_{Hannum} and AA_{Grim}, and the difference increased with advancing age (Supplementary Table S1 and Figure S3, B, D).

Mediation models in all twins

The associations between lifestyle-related factors and AA estimated using the single mediator models are presented in Supplementary Table S2. Higher education was associated with slower AA_{Pheno} and AA_{Grim} but only in the younger twins (B=-0.09, education × age: B=0.09 and B=-0.32, education × age: B=0.13, respectively). Larger BMI was associated with accelerated $AA_{Horvath}$, AA_{Pheno} and AA_{Grim} (B=0.12–0.16). Higher smoking was associated with accelerated AA_{Hannum} only in the older twins (B=0.00, smoking × age: B=0.12), whereas smoking was associated with accelerated AA_{Pheno} and AA_{Grim} also in the younger twins, and the association was stronger in the older twins (B=0.09, smoking × age: B=0.12, and B=0.51, smoking × age: B=0.11, respectively). Greater alcohol use was associated with accelerated AA_{Pheno}, but this was seen only in the older twins (B=0.03; alcohol × age: B=0.09). Furthermore, greater alcohol use was associated with accelerated AA_{Grim} in both age groups (B=0.16). Sport index and leisure index were associated with slower AA_{Grim} in both age groups (B=-0.17 and B=-0.08, respectively). Work index was associated with higher AA_{Grim} especially in the younger twins (B=0.22 and work index × age: B=-0.08, respectively). In all the models, sex was directly associated with epigenetic aging. Male sex was associated with higher $AA_{Horvath}$, AA_{Hannum} and AA_{Grim}, and the association was stronger in the older twins (B=0.09 to 0.15, sex × age: B=0.07 to 0.22). Instead, male sex was associated with slower AA_{Pheno} in the younger twins, but the association turned positive in the older twins (B=-0.11 to -0.08, sex × age: B=0.24 to 0.26).

When the lifestyle factors (education, BMI, smoking, alcohol use, and leisure index) were controlled for each other in the multiple mediator models, higher BMI was still associated with higher $AA_{Horvath}$, AA_{Pheno}, and AA_{Grim} (B=0.11–0.15) (Figure 4). Smoking was associated with accelerated AA_{Hannum} and AA_{Pheno} only in the older twins (B=0.01 and smoking × age: B=0.13 and B=0.06 and smoking × age: B=0.13, respectively), whereas smoking was associated with accelerated AA_{Grim} also in the younger twins, and the association was stronger in the older twins (B=0.47, smoking × age: B=0.14). Greater alcohol use was associated with accelerated AA_{Pheno} only in the older twins (B=0.01; alcohol × age: B=0.06). Education and leisure index were not associated with AA after controlling for other lifestyle-related factors. The direct effect of male sex was still observed on $AA_{Horvath}$, AA_{Hannum} and AA_{Grim}, and the association was stronger in the older twins (B=0.08 to 0.13, sex × age: B=0.07 to 0.14). Instead, male sex was associated with slower AA_{Pheno} in the younger twins, but the association turned positive in the older twins (B=-0.11, sex × age: B=0.23).

Sensitivity analyses

To confirm that observed sex differences in mediator variables and consequently observed indirect effects are not due to the differences in the age distribution between sexes, we re-analyzed the data using polynomial models of age. The interpretation of the results considering sex differences in lifestyle-related factors was very similar to those obtained in the main analysis when a dichotomous variable of age was used (Supplementary Table S3 and Figure S4). Association between age and education followed a quadratic curve. There was a difference in a linear term between sexes (Sex × Age) showing that men were better educated than women in older age. Association between age and BMI followed a quadratic curve, as well. Overall, men had higher level of BMI, and no significant interactions of sex and age were observed. Association between age and (latent) smoking was linear. Men had higher level of smoking and the difference appeared to increase with age, but interaction (Sex × Age) did not reach significance (p=0.066). Association between age and alcohol use followed a cubic curve. There was a difference in both a linear term and quadratic term between sexes (Sex × Age and Sex × Age^2). Overall, men consumed more alcohol than women (Figure S4). The difference was largest at younger age but widened again after 50 years of age. The associations between age and both sport and leisure index were linear. Men had lower level of leisure index, but contrary to our analysis using dichotomous age, there were no significant sex differences in sport index. Association between age and work index followed a quadratic curve and there were no significant sex differences in work index.

The estimation results of the single mediator models using polynomial function of age are presented in Supplementary Table S4. The indirect effects by age were visually inspected using loop plots (Supplementary Figures S5–S17). Interpretation was very similar to the results of the main analysis using a dichotomous variable of age, but there were few exceptions. Education mediated the sex difference only in younger age and only when AA_{Grim} was used to assess epigenetic aging (Supplementary Figure S5). Body mass index did not mediate the sex difference (Supplementary Figure S6). Alcohol use mediated the association only when AA_{Pheno} was used and only in older twins (Supplementary Figure S8).

The estimation results of the multiple mediator models using polynomial function of age are presented in Supplementary Figure S12 and the indirect effects by age in Supplementary Figures S13–S17. These results were very similar to the main results, as well. In line with the main results, also BMI appeared to mediate the sex difference in $AA_{Horvath}$, AA_{Pheno} and AA_{Grim} (Supplementary Figure S14).. This inconsistency between the results of the single and the multiple mediator models of sensitivity analysis is probably due to the fact that insignificant interaction terms were dropped from the multiple mediator models. The only difference to the main analysis was that education did not mediate the association when AA_{Grim} was used to assess epigenetic aging (Supplementary Figure S13).

Mediation models in the opposite-sex twin pairs

In the opposite-sex twin pairs, higher BMI was associated with accelerated $AA_{Horvath}$ (B=0.24) (Supplementary Table S5). Smoking was associated with accelerated AA_{Grim} (B=0.36) and higher sport index with slower $AA_{Horvath}$ (B=-0.14). In all the mediation models, direct association of sex with AA_{Hannum} and AA_{Grim} was observed (B=0.22–0.29). When the lifestyle factors (education, BMI, smoking, alcohol use, and leisure index) were controlled for each other in the multiple mediator models, higher BMI was still associated with accelerated $AA_{Horvath}$ (B=0.26) and smoking with accelerated AA_{Grim} (B=0.35) (Figure 5). Surprisingly, higher leisure index was associated with accelerated AA_{Hannum} (B=0.18). A positive direct effect of male sex on higher AA_{Hannum} and AA_{Grim} was observed (B=0.29 and B=0.19, respectively).

Sex differences in DNAm-based plasma proteins and smoking pack-years

Information on sex is utilized in the estimation of epigenetic age by GrimAge estimator (3). Therefore, the observed sex difference in AA_{Grim} may reflect the estimated sex difference in mortality and not only the differences in DNAm. Also, in DNAm-based surrogates included in the GrimAge estimator the sex difference is in-built, indicating differences between men and women in the actual levels of plasma proteins and smoking pack-years. To further understand the sex differences in biological aging, we also studied the differences in age-adjusted DNAm-based plasma proteins and smoking pack-years in all twins and in the opposite-sex twin pairs. The variables were standardized before the analysis.

For DNAm-based ADM and B2M, a linear model was sufficient (Supplementary Table S6). Men had a lower level of DNAm ADM and B2M in young adulthood, but the difference narrowed or disappeared with age (Supplementary Figure S18, A–B). For DNAm GDF15, a quadratic model was required, and there was a sex difference in the second order term (Supplementary Table S6). Among women the association followed U-shaped pattern, whereas among men the level of DNAm GDF15 decreased linearly with age (Supplementary Figure S18, C). The men had slightly lower level of DNAm GDF15 in younger age. The difference disappeared after 30 years of age but widened again after 50 years of age. Association between age and DNAm cystatin C followed a quadratic curve, and there was a sex difference in the linear term (Supplementary Table S6). The sex difference in this surrogate increased rather steeply from midlife onwards and the men had a higher level especially in older age (Supplementary Figure S18, D). For DNAm leptin the difference in a third order term between the sexes was significant in a cubic model (Supplementary Table S6). Overall, the men had a lower level of DNAm leptin (Supplementary Figure S18, E). In younger age, the sex difference was constant across ages but after 50 years of age the difference slightly narrowed. For DNAm PAI-1 a cubic model was required (Supplementary Table S6). Overall, the men had higher level of DNAm PAI-1, and the sex difference increased rather steeply with age (Supplementary Figure S18, F). For DNAm TIMP-1 and DNAm packyrs a linear model was sufficient (Supplementary Table S6). The sex difference in DNAm TIMP-1 increased with age and the men had a higher level of DNAm TIMP-1 especially in older age (Supplementary Figure S18, G). The men had also a higher level of DNAm-based smoking pack-years, and the difference did not depend on age (Supplementary Figure S18, H).

In the opposite-sex twin pairs, the men had significantly lower levels of DNAm ADM, B2M, and leptin, whereas higher levels of DNAm PAI-1 and DNAm-based smoking pack-years (Supplementary Figure S19).

References

1. Maksimovic J, Phipson B, Oshlack A. A cross-package Bioconductor workflow for analysing methylation array data [version 3; peer review: 4 approved]. *F1000Res*. 2017;5:1281. doi:10.12688/f1000research.8839.3.
2. Fortin JP, Triche TJ, Hansen KD. Preprocessing, normalization and integration of the Illumina HumanMethylationEPIC array with minfi. *Bioinformatics*. 2017;33(4):558-560. doi:10.1093/bioinformatics/btw691.
3. Lu AT, Quach A, Wilson JG, et al. DNA methylation GrimAge strongly predicts lifespan and healthspan. *Aging (Albany NY)*. 2019;11(2):303-327. doi:10.18632/aging.101684.

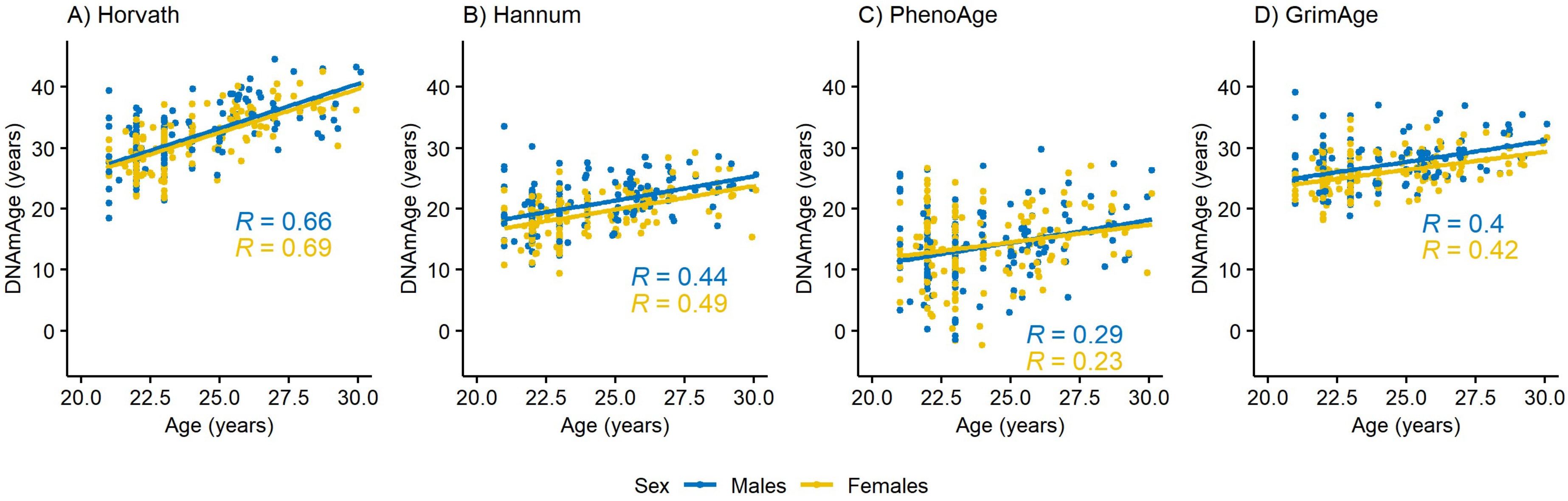

Supplementary Figure S1. Association between chronological age and DNA methylation age (DNAmAge) estimates in opposite-sex (21 to 30 yr-old) twins. R, Pearson's correlation coefficient.

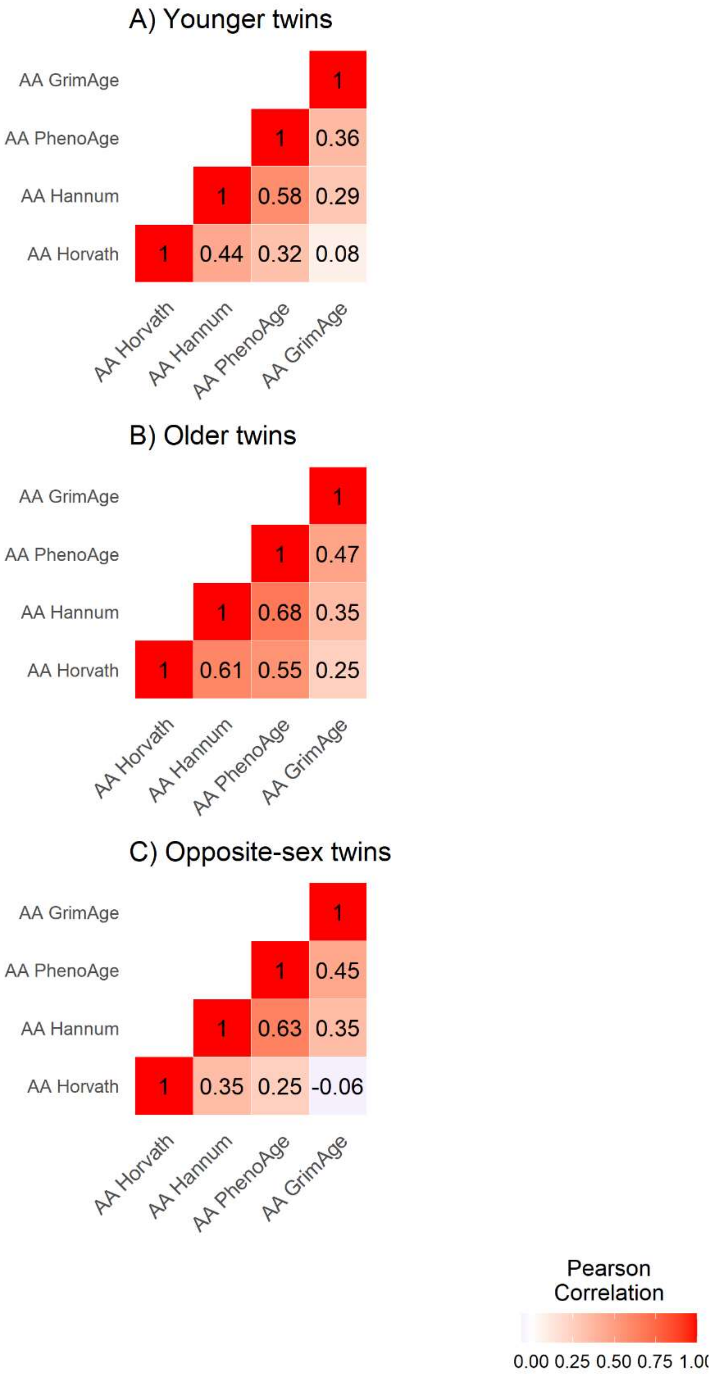

Supplementary Figure S2. Correlation coefficients among epigenetic age acceleration (AA) measures.

Supplementary Table S1. The association between age and epigenetic age acceleration (AA) modelled as a third to first order polynomial function of age (n = 2240).

	Cubic model [a,b,c]			Quadratic model [a,b,c]			Linear model [a,b]		
	B	SE	*p*	B	SE	*p*	B	SE	*p*
Sex (male)									
$AA_{Horvath}$	0.02	0.07	.804	..[d]			..[d]		
AA_{Hannum}	0.22	0.03	<.001	0.22	0.03	<.001	0.22	0.03	<.001
AA_{Pheno}	0.08	0.03	.005	-0.06	0.07	.404	..[d]		
AA_{Grim}	0.27	0.03	<.001	0.27	0.03	<.001	0.28	0.03	<.001
Age									
$AA_{Horvath}$	-0.23	0.09	.012	..[d]			..[d]		
AA_{Hannum}	0.04	0.10	.718	0.03	0.06	.684	-0.01	0.03	.784
AA_{Pheno}	0.10	0.09	.293	0.06	0.07	.360	..[d]		
AA_{Grim}	0.02	0.09	.818	0.04	0.06	.471	-0.03	0.03	.277
Age^2									
$AA_{Horvath}$	-0.97	0.11	<.001	..[d]			..[e]		
AA_{Hannum}	-0.02	0.09	.776	-0.04	0.06	.534			
AA_{Pheno}	-0.03	0.09	.712	-0.20	0.06	.001			
AA_{Grim}	-0.11	0.09	.240	-0.08	0.06	.154			
Age^3									
$AA_{Horvath}$	1.16	0.14	<.001	..[e]			..[e]		
AA_{Hannum}	-0.02	0.14	.872						
AA_{Pheno}	-0.19	0.13	.128						
AA_{Grim}	0.06	0.13	.666						
Sex × Age									
$AA_{Horvath}$	0.02	0.05	.752	..[d]			..[d]		
AA_{Hannum}	0.14	0.03	<.001	0.14	0.03	<.001	0.15	0.03	<.001
AA_{Pheno}	0.23	0.03	<.001	0.16	0.05	.001	..[d]		
AA_{Grim}	0.19	0.04	<.001	0.19	0.04	<.001	0.20	0.04	<.001
Sex × Age^2									
$AA_{Horvath}$	0.16	0.07	.014	..[d]			..[e]		
AA_{Hannum}	..[c]			..[c]					
AA_{Pheno}	..[c]			0.13	0.06	.033			
AA_{Grim}	..[c]			..[c]					
Sex × Age^3									
$AA_{Horvath}$	..[c]			..[e]			..[e]		
AA_{Hannum}	..[c]								
AA_{Pheno}	..[c]								
AA_{Grim}	..[c]								

Notes: B, standardized (STDYX) regression coefficient; SE, standard error.
[a] The model was controlled for zygosity.
[b] SEs were corrected for nested sampling.
[c] The higher order interactions (Sex × Age^2, Sex × Age^3) were freed only when necessary (modification index > 4).
[d] The model was not fitted, because a higher order polynomial model was needed for the AA measure.
[e] The model did not include the corresponding polynomial term.

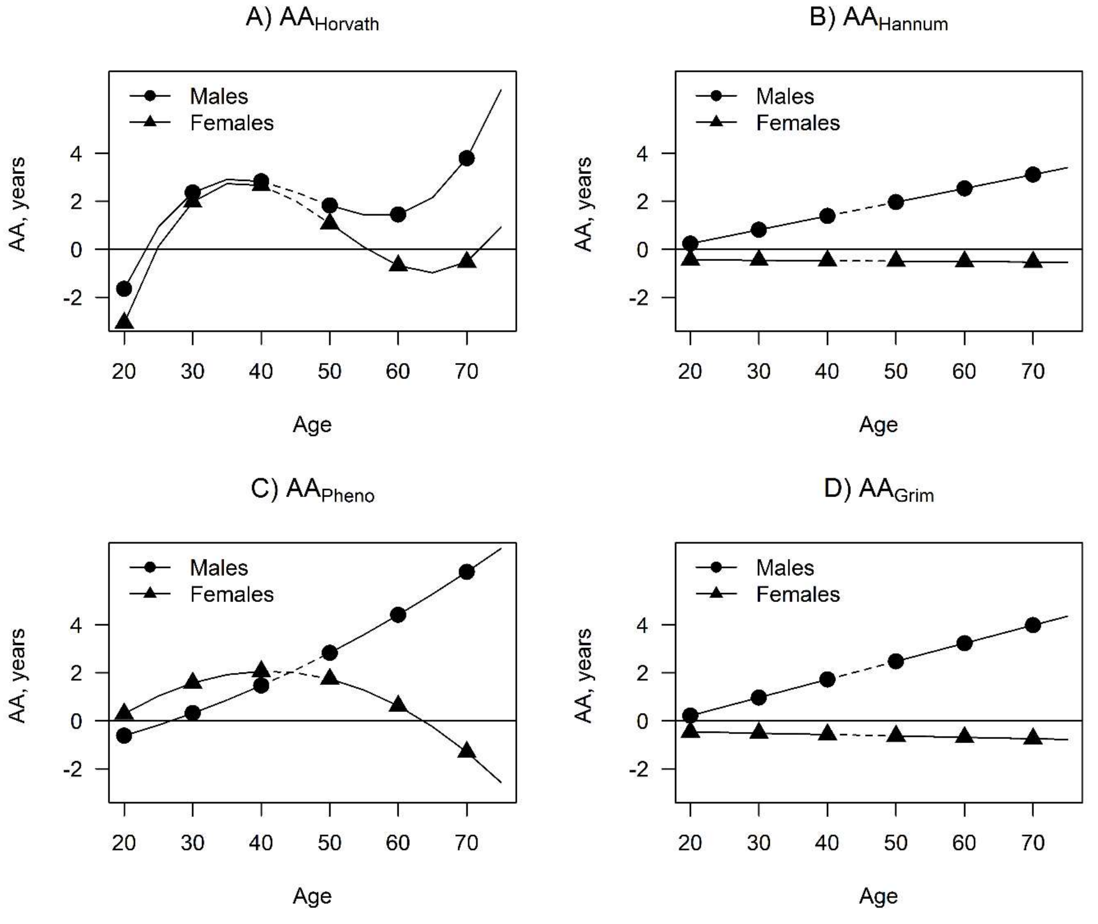

Supplementary Figure S3. Epigenetic age acceleration (AA) by sex and age in the same-sex twins (n = 2240). Dotted line segments denote the lack of data for 43- to 49-year-olds.

Supplementary Table S2. The estimation results of the single mediator models among all twins (n = 2240). Age was treated as dichotomous variable in the modelling

	**Mediator [a,b,c]**													
	**Education**		**Body mass index**		**Smoking**		**Alcohol use**		**Sport index**		**Leisure index**		**Work index**	
	**B (SE)**	***p***	**B (SE)**	***p***	**B (SE)**	***p***	**B (SE)**	***p***	**B (SE)**	***p***	**B (SE)**	***p***	**B (SE)**	***p***
**Epigenetic age acceleration regressed on**														
Mediator (*b*)														
$AA_{Horvath}$	0.04 (0.04)	.317	**0.12 (0.03)**	**<.001**	**-0.10 (0.04)**	**.017**	-0.03 (0.03)	.226	0.03 (0.04)	.439	0.01 (0.04)	.803	0.03 (0.04)	.470
AA_{Hannum}	-0.02 (0.03)	.554	0.03 (0.03)	.327	0.00 (0.04)	.973	0.00 (0.02)	.857	0.01 (0.04)	.846	0.04 (0.04)	.239	0.06 (0.04)	.134
AA_{Pheno}	**-0.09 (0.04)**	**.011**	**0.16 (0.03)**	**<.001**	**0.09 (0.04)**	**.024**	0.03 (0.02)	.125	-0.06 (0.04)	.097	0.03 (0.04)	.523	0.05 (0.04)	.197
AA_{Grim}	**-0.32 (0.03)**	**<.001**	**0.12 (0.03)**	**.002**	**0.11 (0.04)**	**.004**	**0.16 (0.02)**	**<.001**	**-0.17 (0.03)**	**<.001**	**-0.08 (0.03)**	**.019**	**0.22 (0.04)**	**<.001**
Sex (male) (*c*)														
$AA_{Horvath}$	**0.13 (0.03)**	**<.001**	**0.12 (0.03)**	**<.001**	**0.13 (0.03)**	**<.001**	**0.14 (0.03)**	**<.001**	**0.13 (0.03)**	**<.001**	**0.13 (0.03)**	**<.001**	**0.13 (0.03)**	**<.001**
AA_{Hannum}	**0.12 (0.02)**	**<.001**	**0.12 (0.02)**	**.001**	**0.12 (0.03)**	**<.001**	**0.12 (0.03)**	**.001**	**0.12 (0.02)**	**.001**	**0.13 (0.02)**	**<.001**	**0.12 (0.02)**	**<.001**
AA_{Pheno}	**-0.09 (0.03)**	**.001**	**-0.10 (0.03)**	**<.001**	**-0.11 (0.05)**	**.020**	**-0.09 (0.03)**	**<.001**	**-0.08 (0.03)**	**.002**	**-0.08 (0.03)**	**.002**	**-0.08 (0.03)**	**.001**
AA_{Grim}	**0.13 (0.03)**	**<.001**	**0.12 (0.03)**	**<.001**	**0.09 (0.03)**	**<.001**	**0.09 (0.03)**	**.001**	**0.15 (0.03)**	**<.001**	**0.12 (0.03)**	**<.001**	**0.14 (0.03)**	**<.001**
Age (older)														
$AA_{Horvath}$	-0.02 (0.04)	.596	-0.07 (0.03)	.052	**-0.11 (0.04)**	**.017**	-0.03 (0.03)	.426	-0.03 (0.03)	.385	0.08 (0.16)	.608	-0.01 (0.04)	.729
AA_{Hannum}	0.02 (0.04)	.619	-0.02 (0.04)	.656	-0.05 (0.04)	.191	0.02 (0.04)	.579	0.00 (0.04)	.998	0.01 (0.03)	.894	0.01 (0.04)	.898
AA_{Pheno}	**-0.10 (0.05)**	**.03**	**-0.16 (0.04)**	**<.001**	-0.13 (0.06)	.030	-0.07 (0.04)	.051	**-0.10 (0.03)**	**.003**	**-0.11 (0.03)**	**.002**	-0.10 (0.04)	.447
AA_{Grim}	**-0.16 (0.04)**	**<.001**	**-0.08 (0.03)**	**.018**	**0.10 (0.03)**	**.004**	0.00 (0.03)	.936	-0.01 (0.03)	.790	-0.04 (0.03)	.218	0.01 (0.03)	.811
Sex (male)×Age (older) (*i2*)														
$AA_{Horvath}$	**0.08 (0.03)**	**.007**	**0.09 (0.03)**	**.011**	**0.07 (0.03)**	**.019**	**0.08 (0.03)**	**.008**	**0.09 (0.03)**	**.006**	**0.08 (0.03)**	**.010**	**0.08 (0.03)**	**.011**
AA_{Hannum}	**0.13 (0.03)**	**<.001**	**0.14 (0.03)**	**<.001**	**0.12 (0.03)**	**<.001**	**0.13 (0.03)**	**<.001**	**0.14 (0.03)**	**<.001**	**0.14 (0.03)**	**<.001**	**0.14 (0.03)**	**<.001**
AA_{Pheno}	**0.25 (0.03)**	**<.001**	**0.26 (0.03)**	**<.001**	**0.24 (0.04)**	**<.001**	**0.26 (0.04)**	**<.001**	**0.25 (0.03)**	**<.001**	**0.26 (0.03)**	**<.001**	**0.25 (0.03)**	**<.001**
AA_{Grim}	**0.22 (0.04)**	**<.001**	**0.20 (0.04)**	**<.001**	**0.12 (0.03)**	**.001**	**0.19 (0.04)**	**<.001**	**0.18 (0.04)**	**<.001**	**0.19 (0.04)**	**<.001**	**0.19 (0.04)**	**<.001**

Mediator×Age (older) (*i3*)														
$AA_{Horvath}$	-0.03 (0.04)	.496	-0.02 (0.04)	.566	**0.11 (0.04)**	**.008**	0.01 (0.03)	.834	-0.01 (0.04)	.777	-0.03 (0.03)	.458	0.03 (0.04)	.526
AA_{Hannum}	0.05 (0.04)	.294	0.02 (0.04)	.641	**0.12 (0.03)**	**<.001**	0.05 (0.03)	.093	0.00 (0.04)	.999	-0.03 (0.04)	.482	-0.05 (0.04)	.221
AA_{Pheno}	**0.09 (0.05)**	**.050**	-0.04 (0.04)	.279	**0.12 (0.04)**	**.001**	**0.09 (0.03)**	**.002**	0.02 (0.05)	.602	0.00 (0.05)	.983	-0.04 (0.04)	.419
AA_{Grim}	**0.13 (0.04)**	**.002**	-0.01 (0.03)	.877	**0.11 (0.04)**	**.004**	0.03 (0.03)	.349	0.00 (0.04)	.962	-0.04 (0.04)	.367	-0.08 (0.04)	.064
**Mediator regressed on**														
Sex (male) (*a*)	-0.02 (0.02)	.270	**0.09 (0.03)**	**.001**	**0.09 (0.05)**	**.035**	**0.29 (0.03)**	**<.001**	**0.08 (0.03)**	**.014**	**-0.13 (0.03)**	**<.001**	-0.01 (0.03)	.781
Age (older)	**-0.71 (0.02)**	**<.001**	**0.39 (0.03)**	**<.001**	**-0.32 (0.04)**	**<.001**	**-0.11 (0.02)**	**<.001**	**0.13 (0.05)**	**.003**	-0.07 (0.05)	.142	**-0.27 (0.05)**	**<.001**
Sex×Age (older) (*i1*)	**0.15 (0.03)**	**<.001**	-0.03 (0.03)	.362	**0.11 (0.05)**	**.024**	-0.03 (0.03)	.248	-0.07 (0.04)	.052	-0.01 (0.04)	.890	0.06 (0.04)	.147

Notes: B, standardized (STDYX) regression coefficient; SE, standard error; AA, epigenetic age acceleration.

[a] The model was controlled for zygosity.

[b] SEs were corrected for nested sampling.

[c] Significant regression coefficients at the level .05 are presented in bold

Supplementary Table S3. Sensitivity analysis: The association between age and mediators modelled as a third to first order polynomial function of age (n = 2240).

	Cubic model [a,b,c]			Quadratic model [a,b,c]			Linear model [a,b]		
	B	SE	*p*	B	SE	*p*	B	SE	*p*
Sex (male)									
Education	0.06	0.07	.022	0.05	0.02	.024	..[d]		
Body mass index	0.07	0.03	.007	0.07	0.03	.008	..[d]		
Smoking	0.11	0.04	.003	0.14	0.03	<.001	0.15	0.03	<.001
Alcohol use	0.10	0.07	.150	..[d]			..[d]		
Sport index	0.04	0.03	.040	0.03	0.23	.146	0.04	0.03	.259
Leisure index	-0.13	0.03	<.001	-0.13	0.03	<.001	-0.13	0.03	<.001
Work index	0.02	0.03	.545	0.02	0.03	.532	..[d]		
Age									
Education	-0.67	0.06	<.001	-0.63	0.46	<.001	..[d]		
Body mass index	0.48	0.08	<.001	0.55	0.06	<.001	..[d]		
Smoking	0.26	0.33	.429	-0.01	0.20	.948	-0.35	0.07	<.001
Alcohol use	-0.02	0.06	.795	..[d]			..[d]		
Sport index	0.24	0.14	.084	0.08	0.07	.310	0.12	0.05	.011
Leisure index	-0.05	0.15	.746	-0.14	0.07	.052	-0.10	0.05	.051
Work index	0.05	0.16	.756	-0.10	0.07	.139	..[d]		
Age^2									
Education	0.10	0.10	.061	-0.08	0.05	.050	..[e]		
Body mass index	-0.25	0.10	.013	-0.16	0.06	.007			
Smoking	0.18	0.20	.361	-0.25	0.14	.084			
Alcohol use	0.10	0.08	.219	..[d]					
Sport index	0.19	0.13	.147	0.08	0.09	.395			
Leisure index	0.14	0.12	.231	0.08	0.09	.369			
Work index	-0.16	0.11	.144	-0.27	0.09	.003			
Age^3									
Education	0.10	0.10	.313	..[e]			..[e]		
Body mass index	0.16	0.13	.207						
Smoking	-0.79	0.43	.063						
Alcohol use	-0.20	0.10	.036						
Sport index	-0.32	0.25	.193						
Leisure index	-0.18	0.24	.471						
Work index	-0.29	0.26	.256						
Sex × Age									
Education	0.12	0.03	<.001	0.11	0.03	<.001	..[d]		
Body mass index	-0.02	0.03	.526	-0.03	0.03	.369	..[d]		
Smoking	0.02	0.06	.776	0.10	0.05	.046	0.10	0.05	.066
Alcohol use	-0.14	0.05	.004	..[d]					
Sport index	-0.06	0.04	.091	-0.05	0.04	.122	-0.06	0.04	.111
Leisure index	0.00	0.04	.010	0.01	0.04	.878	0.00	0.04	.908
Work index	0.03	0.04	.465	0.03	0.04	.398	..[d]		
Sex × Age^2									

Education	..[c]			..[c]			..[e]		
Body mass index	..[c]			..[c]					
Smoking	..[c]			..[c]					
Alcohol use	0.14	0.07	.032	..[d]					
Sport index	..[c]			..[c]					
Leisure index	..[c]			..[c]					
Work index	..[c]			..[c]					
Sex × Age^3									
Education	..[c]			..[e]			..[e]		
Body mass index	..[c]								
Smoking	..[c]								
Alcohol use	..[c]								
Sport index	..[c]								
Leisure index	..[c]								
Work index	..[c]								

Notes: B, standardized regression coefficient; SE, standard error.
[a] The model was controlled for zygosity.
[b] SEs were corrected for nested sampling.
[c] The higher order interactions (Sex × Age^2, Sex × Age^3) were freed only when necessary (modification index > 4).
[d] The model was not fitted, because a higher order polynomial model was needed for the mediator.
[e] The model did not include the corresponding polynomial term.

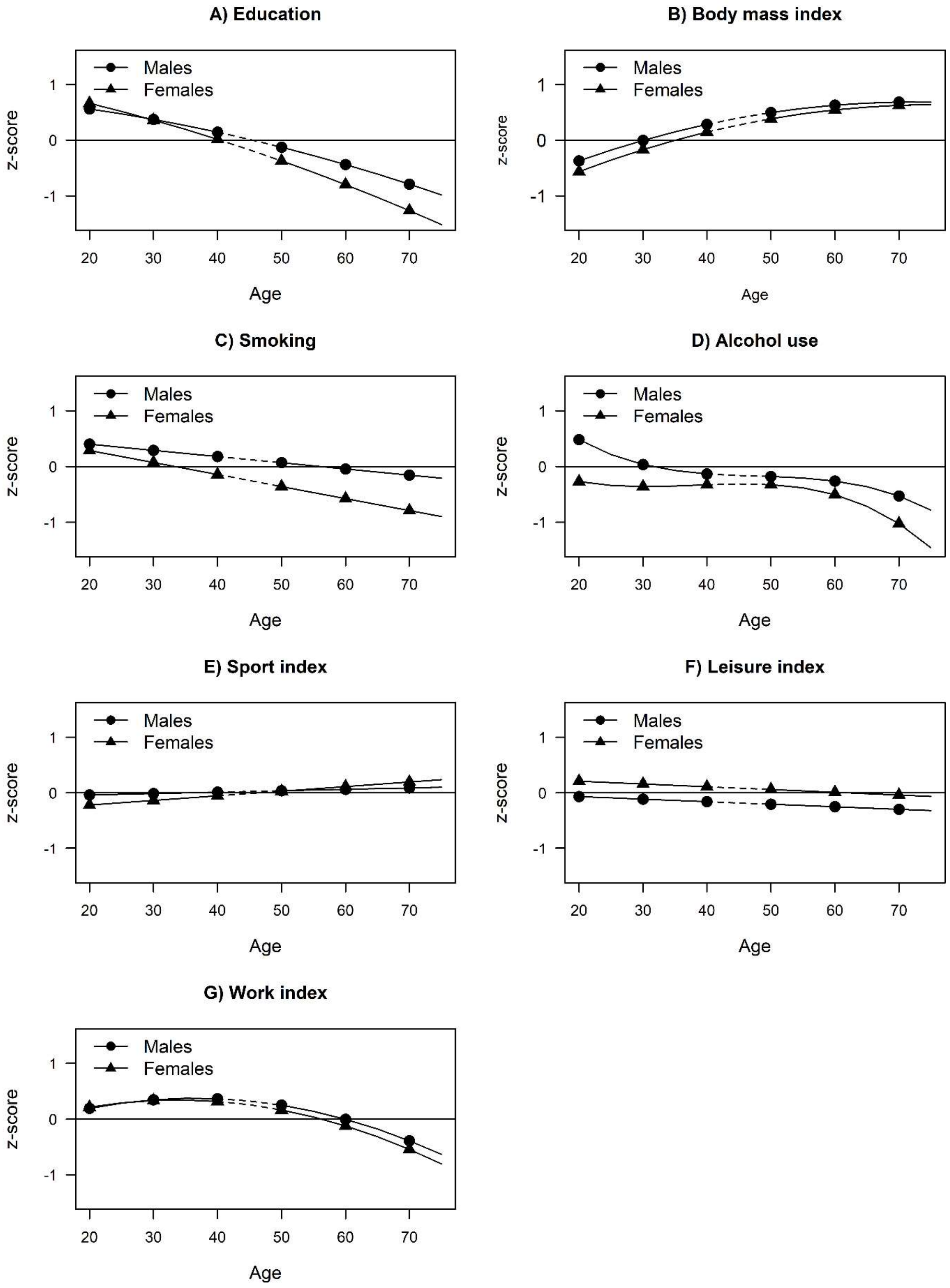

Supplementary Figure S4. Sensitivity analysis: Mediators by sex and age in all twins (n = 2240). Dotted line segments denote the lack of data for 43- to 49-year-olds.

Supplementary Table S4. Sensitivity analysis: The estimation results of the single mediator models among all twins (n = 2240).
Polynomial functions of age are used in the modelling.

	**Mediator [a,b,c]**													
	**Education**		**Body mass index**		**Smoking**		**Alcohol use**		**Sport index**		**Leisure index**		**Work index**	
	**B (SE)**	***p***	**B (SE)**	***p***	**B (SE)**	***p***	**B (SE)**	***p***	**B (SE)**	***p***	**B (SE)**	***p***	**B (SE)**	***p***
**Epigenetic age acceleration regressed on**														
Mediator														
$AA_{Horvath}$	0.05 (0.03)	.101	**0.07 (0.03)**	**.004**	-0.04 (0.04)	.243	-0.01 (0.03)	.700	0.03 (0.03)	.342	0.01 (0.03)	.871	0.05 (0.03)	.123
AA_{Hannum}	-0.01 (0.03)	.839	0.04 (0.03)	.116	**0.09 (0.03)**	**.006**	0.04 (0.03)	.155	0.01 (0.03)	.799	0.02 (0.04)	.53	0.04 (0.03)	.209
AA_{Pheno}	-0.06 (0.03)	.055	**0.15 (0.03)**	**<.001**	**0.18 (0.03)**	**<.001**	**0.10 (0.03)**	**<.001**	-0.04 (0.04)	.261	0.03 (0.04)	.447	0.04 (0.03)	.308
AA_{Grim}	**-0.25 (0.03)**	**<.001**	**0.12 (0.03)**	**<.001**	**0.64 (0.02)**	**<.001**	**0.19 (0.03)**	**<.001**	**-0.17 (0.03)**	**<.001**	**-0.11 (0.03)**	**.001**	**0.19 (0.03)**	**<.001**
Sex (male)														
$AA_{Horvath}$	0.01 (0.07)	.893	0.02 (0.07)	.825	**0.17 (0.02)**	**<.001**	0.02 (0.07)	.789	0.12 (0.07)	.846	0.02 (0.07)	.818	0.02 (0.07)	.829
AA_{Hannum}	**0.22 (0.03)**	**<.001**	**0.22 (0.03)**	**<.001**	**0.20 (0.03)**	**<.001**	**0.21 (0.03)**	**<.001**	**0.22 (0.03)**	**<.001**	**0.22 (0.03)**	**<.001**	**0.22 (0.03)**	**<.001**
AA_{Pheno}	-0.05 (0.07)	.498	-0.07 (0.07)	.320	**0.07 (0.03)**	**0.019**	0.06 (0.03)	.041	-0.06 (0.07)	.435	**-0.06 (0.07)**	**.432**	-0.05 (0.07)	.437
AA_{Grim}	**0.28 (0.03)**	**<.001**	**0.27 (0.03)**	**<.001**	**0.17 (0.02)**	**<.001**	**0.23 (0.03)**	**<.001**	**0.28 (0.03)**	**<.001**	**0.26 (0.03)**	**<.001**	**0.27 (0.03)**	**<.001**
Age														
$AA_{Horvath}$	-0.53 (0.49)	.285	0.09 (0.67)	.889	**0.62 (0.23)**	**.007**	-0.30 (0.32)	.348	**-0.29 (0.13)**	**.023**	**-0.31 (0.12)**	**.013**	-0.12 (0.12)	.334
AA_{Hannum}	0.41 (0.50)	.404	0.28 (0.66)	.671	-0.03 (0.03)	.306	0.46 (0.30)	.129	-0.02 (0.13)	.884	-0.09 (0.12)	.440	-0.06 (0.12)	.597
AA_{Pheno}	0.56 (0.53)	.287	-0.60 (0.65)	.355	**-0.13 (0.04)**	**.001**	**0.80 (0.29)**	**.006**	0.11 (0.14)	.457	0.06 (0.14)	.699	-0.01 (0.13)	.961
AA_{Grim}	**1.23 (0.47)**	**.008**	-0.26 (0.59)	.663	**0.15 (0.04)**	**<.001**	0.26 (0.34)	.467	-0.04 (0.13)	.734	-0.16 (0.11)	.153	-0.19 (0.12)	.101
Age^2														
$AA_{Horvath}$	**-0.98 (0.11)**	**<.001**	**-0.96 (0.11)**	**<.001**	**-0.58 (0.17)**	**.001**	**-0.97 (0.12)**	**<.001**	**-0.98 (0.11)**	**<.001**	**-0.98 (0.11)**	**<.001**	**-0.96 (0.11)**	**<.001**
AA_{Hannum}	..[d]		..[d]		..[d]		..[d]		..[d]		..[d]		..[d]	
AA_{Pheno}	**-0.18 (0.07)**	**.007**	**-0.17 (0.06)**	**.007**	..[d]		**-0.13 (0.06)**	**.019**	**-0.20 (0.05)**	**.002**	**-0.2 (0.06)**	**.001**	**-0.2 (0.06)**	**.002**
AA_{Grim}	..[d]		..[d]		..[d]		..[d]		..[d]		..[d]		..[d]	
Age^3														
$AA_{Horvath}$	**1.16 (0.14)**	**<.001**	**1.15 (0.14)**	**<.001**	..[d]		**1.16 (0.14)**	**<.001**	**1.16 (0.14)**	**<.001**	**1.16 (0.14)**	**<.001**	**1.19 (0.15)**	**<.001**
AA_{Hannum}	..[d]		..[d]		..[d]		..[d]		..[d]		..[d]		..[d]	

AA_{Pheno}	..[d]		..[d]		..[d]		..[d]		..[d]		..[d]		..[d]	
AA_{Grim}	..[d]		..[d]		..[d]		..[d]		..[d]		..[d]		..[d]	
Sex (male)×Age														
$AA_{Horvath}$	0.01 (0.05)	.877	0.02 (0.05)	.721	0.00 (0.04)	.980	0.02 (0.05)	.741	0.02 (0.05)	.744	0.01 (0.05)	.842	0.01 (0.05)	.808
AA_{Hannum}	**0.14 (0.03)**	**<.001**	**0.14 (0.03)**	**<.001**	**0.11 (0.03)**	**<.001**	**0.13 (0.03)**	**<.001**	**0.15 (0.03)**	**<.001**	**0.14 (0.03)**	**<.001**	**0.14 (0.03)**	**<.001**
AA_{Pheno}	**0.17 (0.05)**	**<.001**	**0.16 (0.05)**	**<.001**	**0.22 (0.03)**	**<.001**	**0.22 (0.03)**	**<.001**	**0.16 (0.05)**	**<.035**	**0.16 (0.05)**	**.001**	**0.16 (0.05)**	**.001**
AA_{Grim}	**0.22 (0.04)**	**<.001**	**0.20 (0.04)**	**<.001**	**0.08 (0.02)**	**<.001**	**0.21 (0.04)**	**<.001**	**0.19 (0.04)**	**<.001**	**0.20 (0.04)**	**<.001**	**0.20 (0.04)**	**<.001**
Sex (male)×Age^2														
$AA_{Horvath}$	**0.17 (0.07)**	**.010**	**0.16 (0.07)**	**.015**	..[d]		**0.16 (0.07)**	**.014**	**0.16 (0.07)**	**.013**	**0.16 (0.07)**	**.013**	**0.16 (0.07)**	**.015**
AA_{Hannum}	..[d]		..[d]		..[d]		..[d]		..[d]		..[d]		..[d]	
AA_{Pheno}	**0.12 (0.06)**	**.050**	**0.13 (0.06)**	**.030**	..[d]		..[d]		**0.13 (0.06)**	**.035**	**0.14 (0.06)**	**.032**	**0.13 (0.06)**	**.038**
AA_{Grim}	..[d]		..[d]		..[d]		..[d]		..[d]		..[d]		..[d]	
Sex (male)×Age^3														
$AA_{Horvath}$	..[d]		..[d]		..[d]		..[d]		..[d]		..[d]		..[d]	
AA_{Hannum}	..[d]		..[d]		..[d]		..[d]		..[d]		..[d]		..[d]	
AA_{Pheno}	..[d]		..[d]		..[d]		..[d]		..[d]		..[d]		..[d]	
AA_{Grim}	..[d]		..[d]		..[d]		..[d]		..[d]		..[d]		..[d]	
Mediator×Age														
$AA_{Horvath}$	-0.35 (0.50)	.486	0.35 (0.66)	.594	-0.04 (0.08)	.573	-0.08 (0.32)	.803	-0.07 (0.11)	.524	-0.10 (0.10)	.303	0.14 (0.11)	.222
AA_{Hannum}	0.43 (0.49)	.384	0.31 (0.67)	.645	**0.11 (0.03)**	**<.001**	0.46 (0.30)	.122	0.00 (0.13)	.947	-0.09 (0.12)	.447	-0.07 (0.11)	.511
AA_{Pheno}	0.56 (0.54)	.298	-0.57 (0.64)	.372	**0.12 (0.03)**	**.001**	**0.78 (0.29)**	**.006**	0.04 (0.13)	.758	-0.01 (0.14)	.929	-0.08 (0.12)	.490
AA_{Grim}	**1.43 (0.46)**	**.002**	-0.18 (0.59)	.761	**0.19 (0.02)**	**<.001**	0.26 (0.34)	.448	-0.04 (0.13)	.773	-0.13 (0.11)	.267	**-0.23 (0.11)**	**.033**
**Mediator regressed on**														
Sex (male)	**0.05 (0.02)**	**.024**	**0.07 (0.03)**	**.008**	**0.14 (0.03)**	**<.001**	0.10 (0.07)	.147	0.04 (0.03)	.248	**-0.13 (0.03)**	**<.001**	0.02 (0.03)	.467
Age	**-0.63 (0.05)**	**<.001**	**0.55 (0.06)**	**<.001**	**-0.41 (0.05)**	**<.001**	-0.02 (0.06)	.794	**0.12 (0.05)**	**.013**	**-0.10 (0.05)**	**.050**	-0.11 (0.07)	.113
Age^2	**-0.08 (0.04)**	**.050**	**-0.16 (0.06)**	**.007**	..[d]		0.10 (0.08)	.216	..[d]		..[d]		**-0.27 (0.09)**	**.002**
Age^3	.. ..[d]		.. ..[d]		..[d]		**-0.20 (0.10)**	**.036**	..[d]		..[d]		..[d]	
Sex×Age	**0.11 (0.03)**	**<.001**	-0.03 (0.03)	.369	**0.12 (0.04)**	**.001**	**-0.14 (0.05)**	**.004**	-0.05 (0.04)	.117	0.01 (0.04)	.900	0.04 (0.04)	.348
Sex×Age^2	.. ..[d]		.. ..[d]		..[d]		**0.14 (0.07)**	**.033**	..[d]		..[d]		..[d]	

Notes: B, standardized (STDYX) regression coefficient; SE, standard error; AA, epigenetic age acceleration.
[a] The model was controlled for zygosity.
[b] SEs were corrected for nested sampling.
[c] Significant regression coefficients at the level .05 are presented in bold
[d] The parameter was estimated only when necessary (modification index > 4).

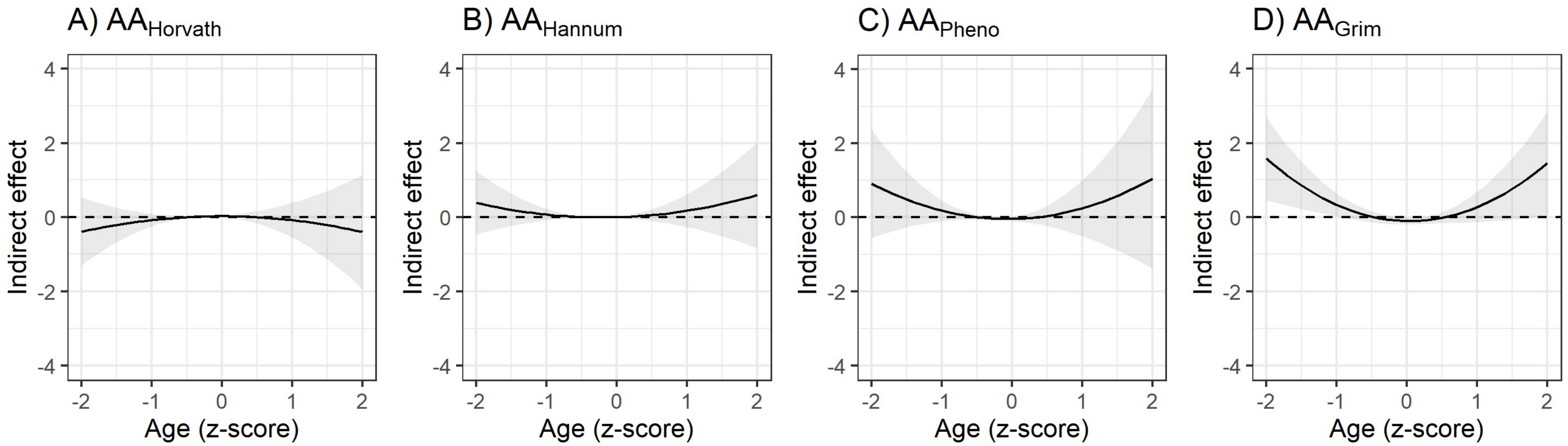

Supplementary Figure S5. Sensitivity analysis: Indirect effects of sex (male) on epigenetic age acceleration (AA) through education by age (single mediator models).

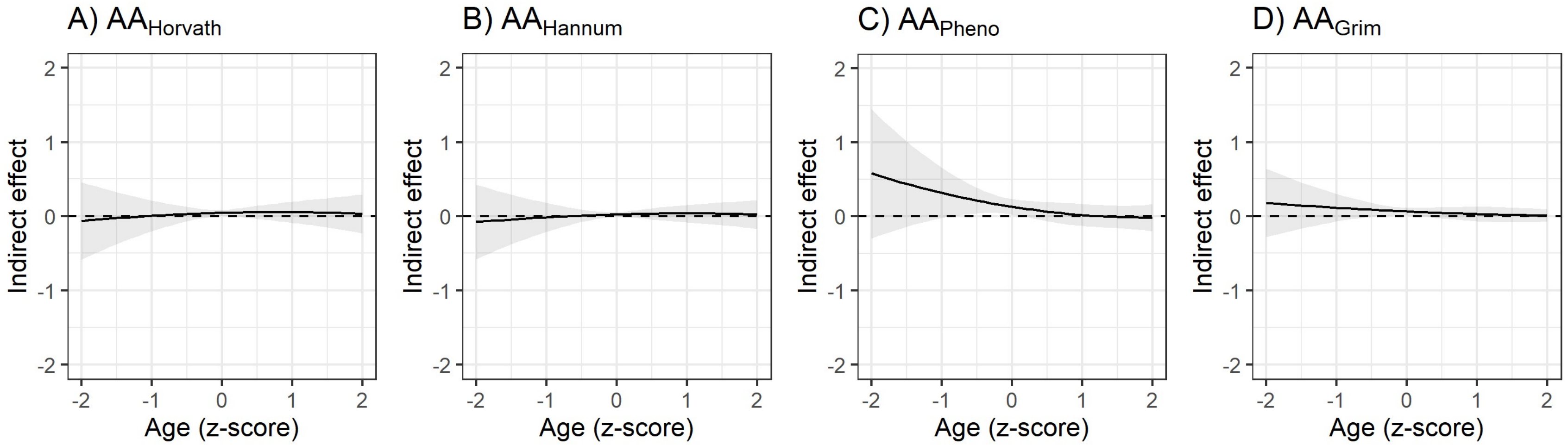

Supplementary Figure S6. Sensitivity analysis: Indirect effects of sex (male) on epigenetic age acceleration (AA) through body mass index by age (single mediator models).

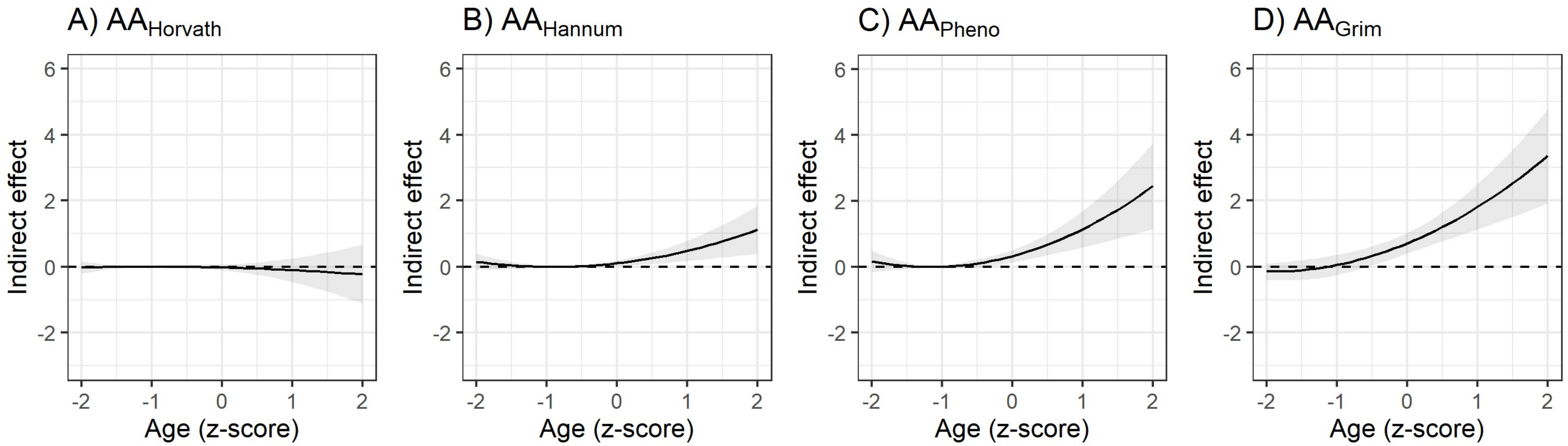

Supplementary Figure S7. Sensitivity analysis: Indirect effects of sex (male) on epigenetic age acceleration (AA) through smoking by age (single mediator models).

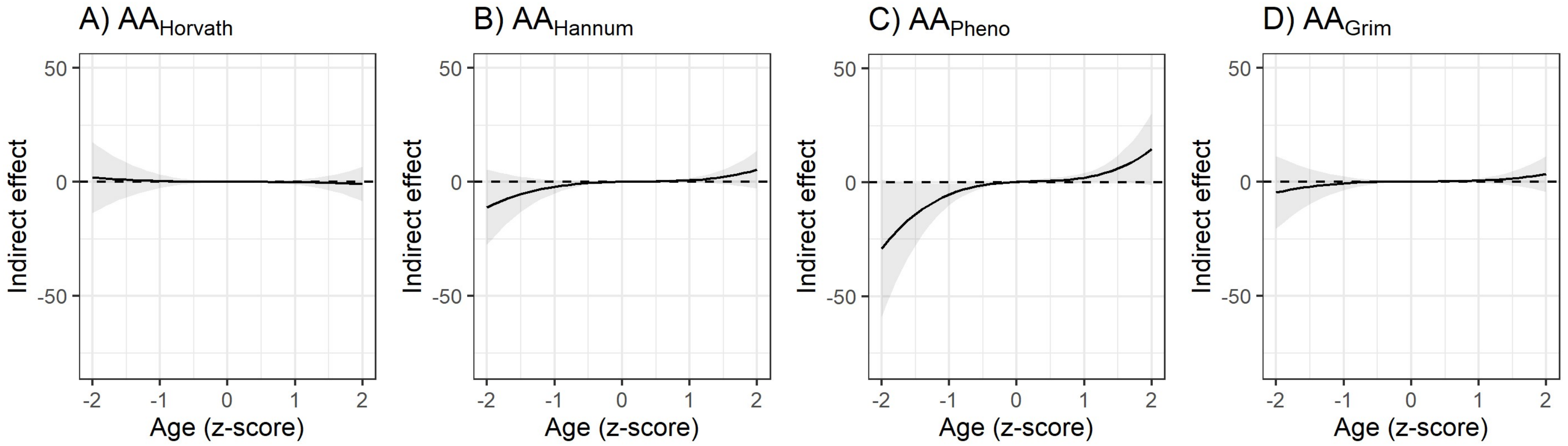

Supplementary Figure S8. Sensitivity analysis: Indirect effects of sex (male) on epigenetic age acceleration (AA) through alcohol use by age (single mediator models).

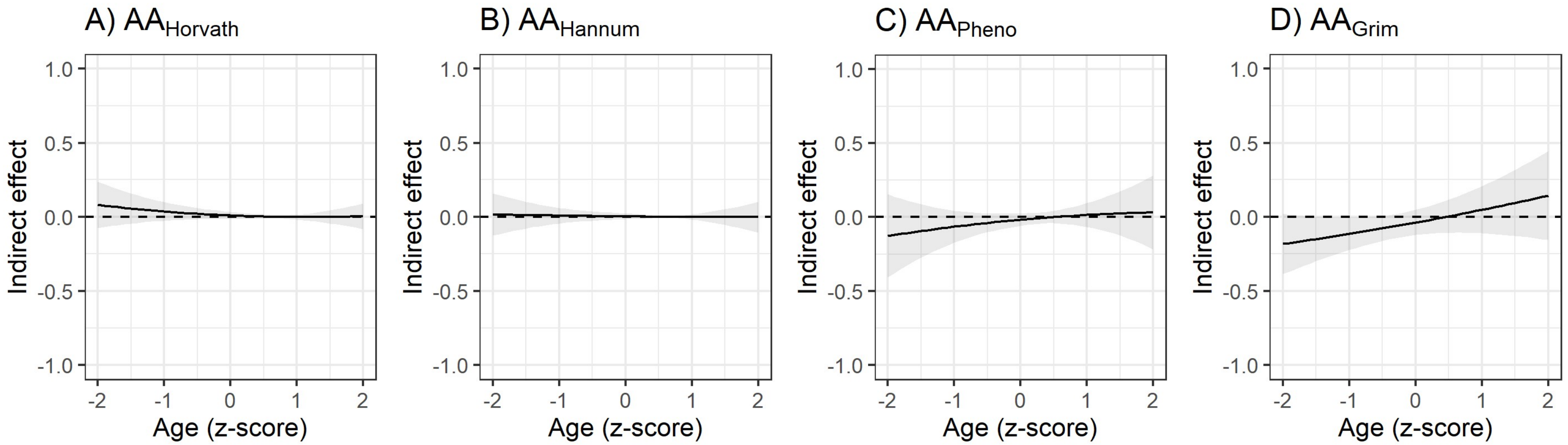

Supplementary Figure S9. Sensitivity analysis: Indirect effects of sex (male) on epigenetic age acceleration (AA) through sport index by age (single mediator models).

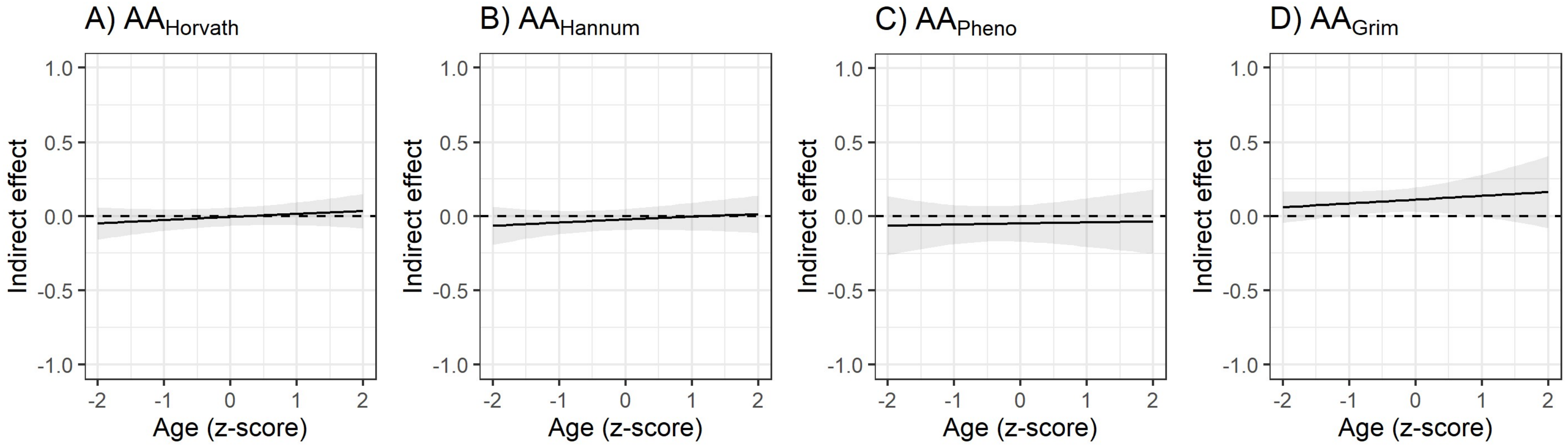

Supplementary Figure S10. Sensitivity analysis: Indirect effects of sex (male) on epigenetic age acceleration (AA) through leisure index by age (single mediator models).

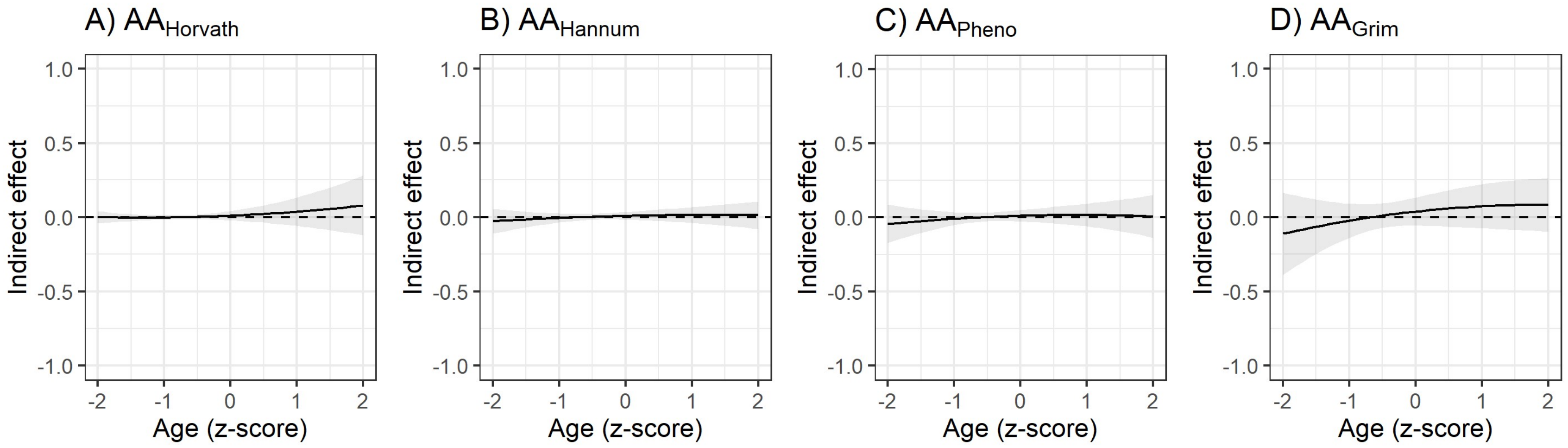

Supplementary Figure S11. Sensitivity analysis: Indirect effects of sex (male) on epigenetic age acceleration (AA) through work index by age (single mediator models).

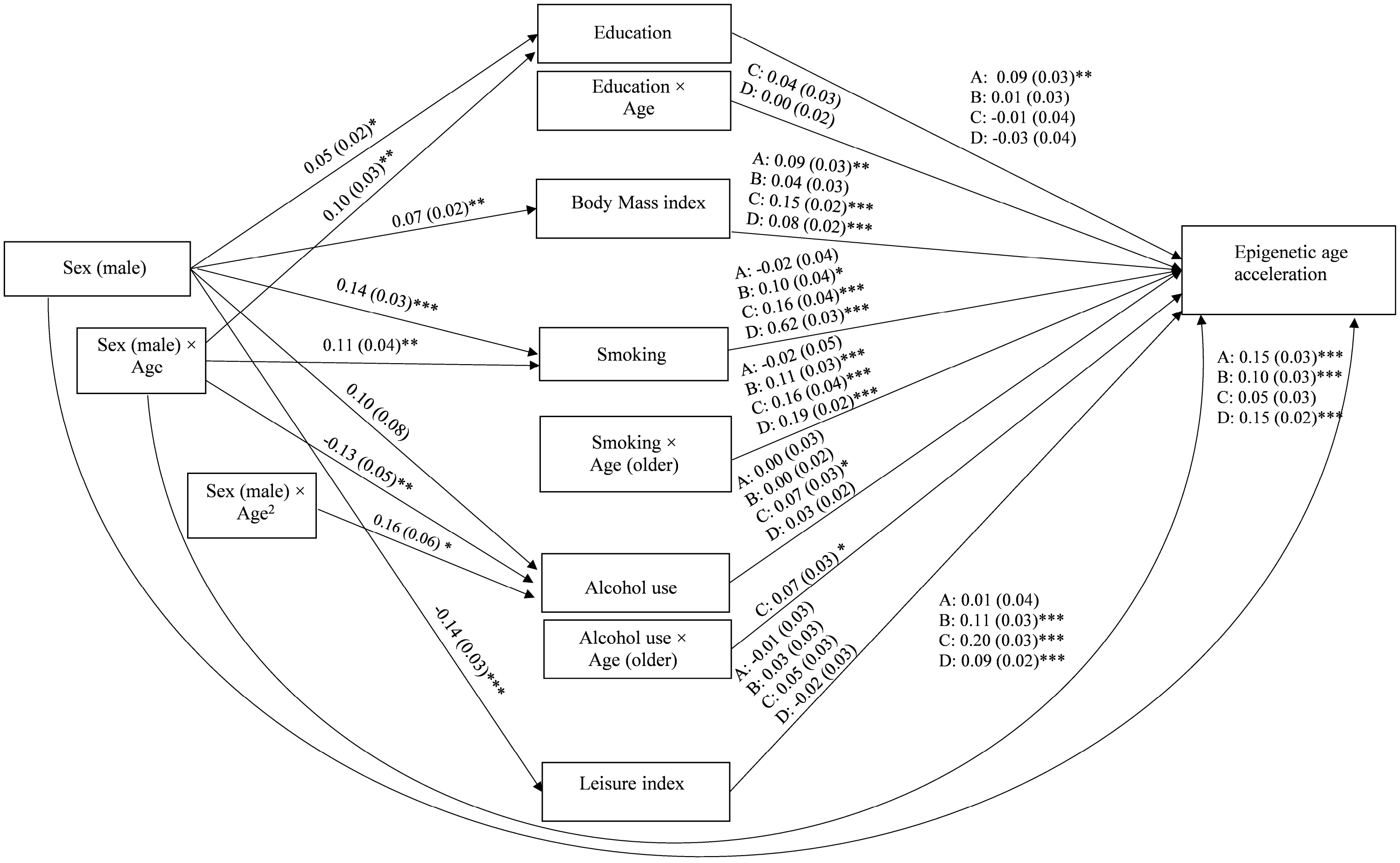

Supplementary Figure S12. Sensitivity analysis: The path diagram of the multiple mediator model in all twins (n = 2240).
Standardized regression coefficients (standard errors) are presented. Polynomial functions of continuous age were used in the modelling. The modelling was conducted separately for each epigenetic age acceleration (AA) measure: A, $AA_{Horvath}$; B, AA_{Hannum}; C, AA_{Pheno}; D, AA_{Grim}. ***, $p < .001$; **, $p < .01$; *, $p < .05$.

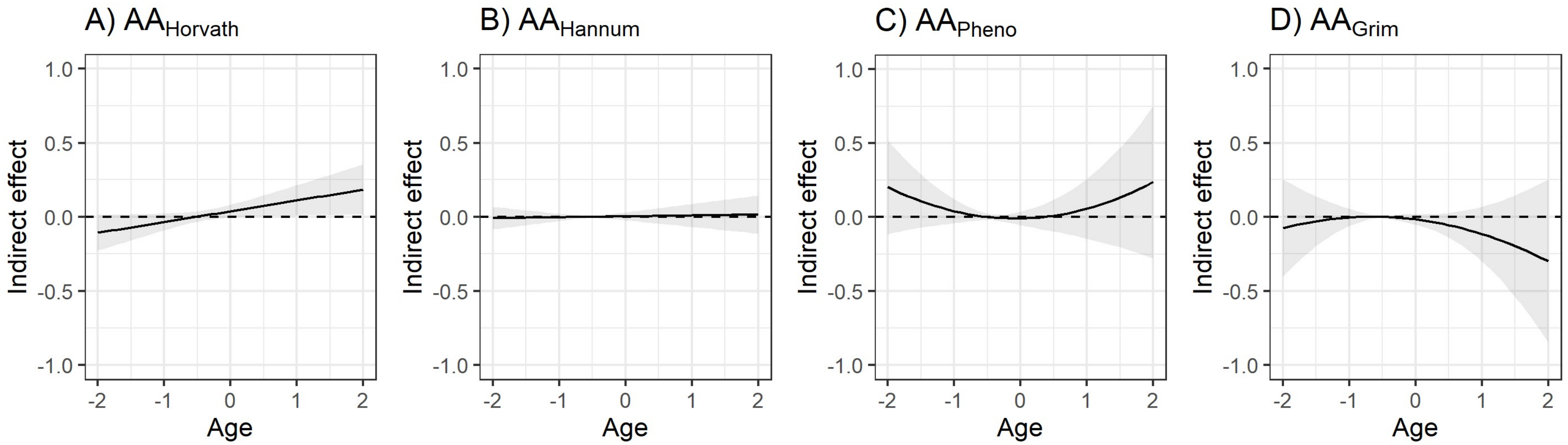

Supplementary Figure S13. Sensitivity analysis: Indirect effects of sex (male) on epigenetic age acceleration (AA) through education by age (multiple mediator models).

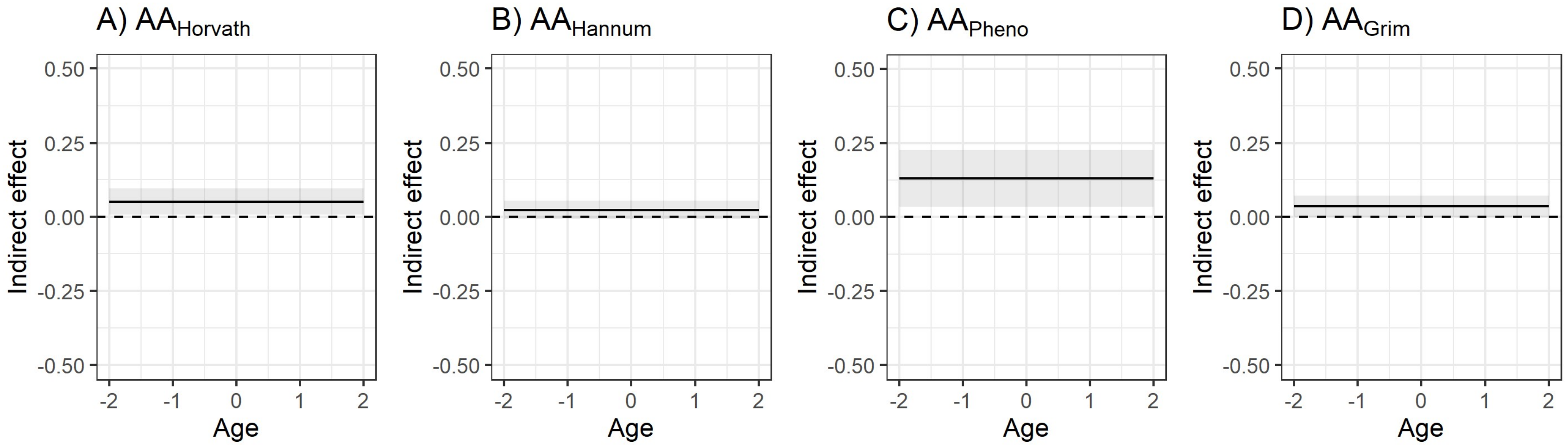

Supplementary Figure S14. Sensitivity analysis: Indirect effects of sex (male) on epigenetic age acceleration (AA) through body mass index by age (multiple mediator models).

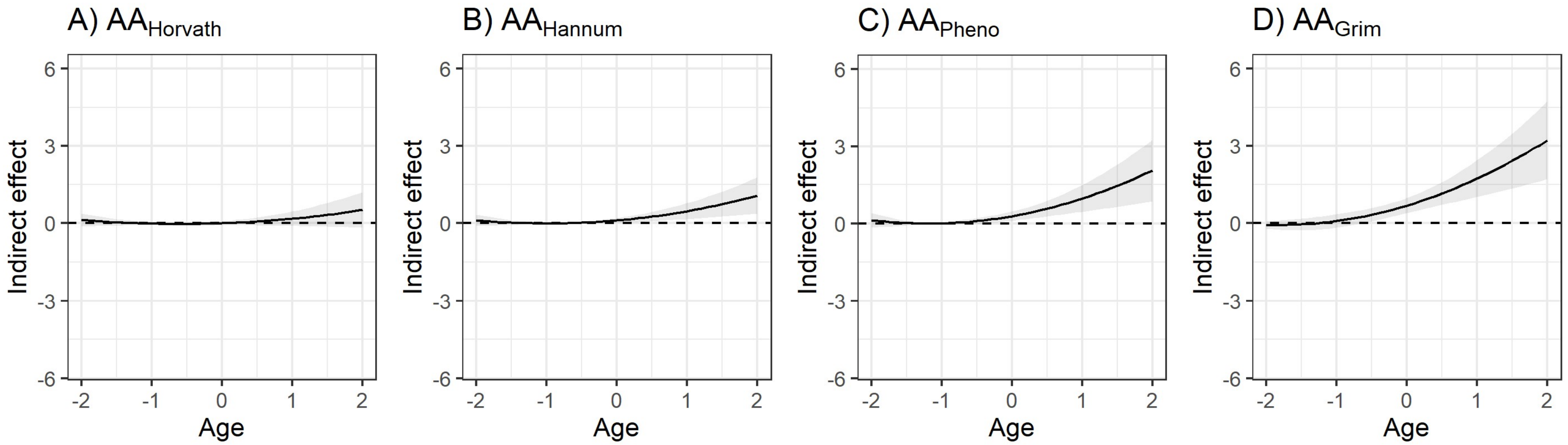

Supplementary Figure S15. Sensitivity analysis: Indirect effects of sex (male) on epigenetic age acceleration (AA) through smoking by age (multiple mediator models).

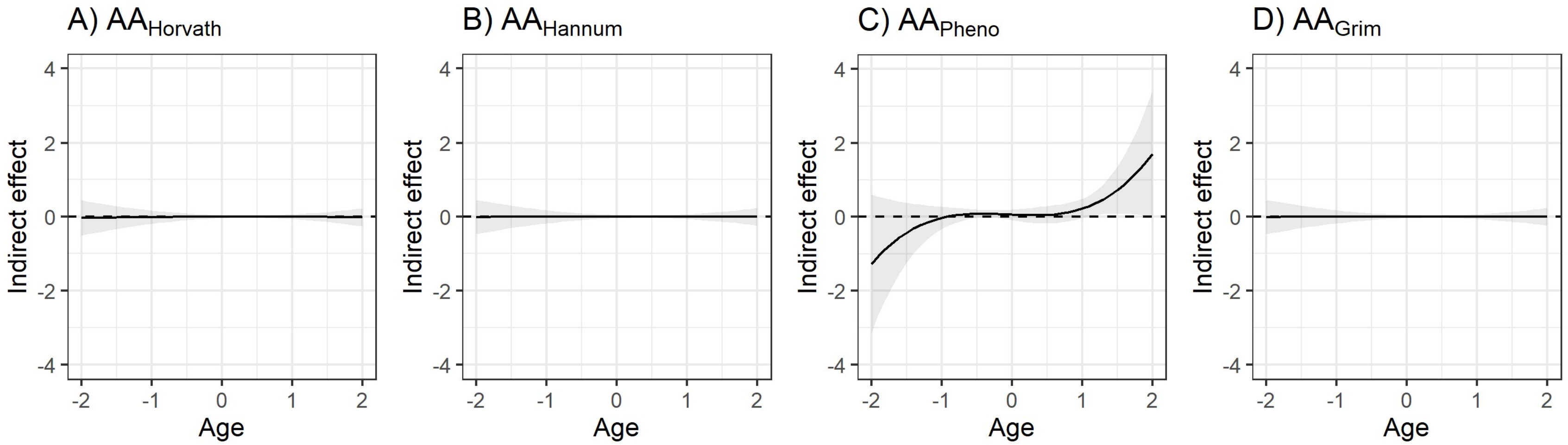

Supplementary Figure S16. Sensitivity analysis: Indirect effects of sex (male) on epigenetic age acceleration (AA) through alcohol use by age (multiple mediator models).

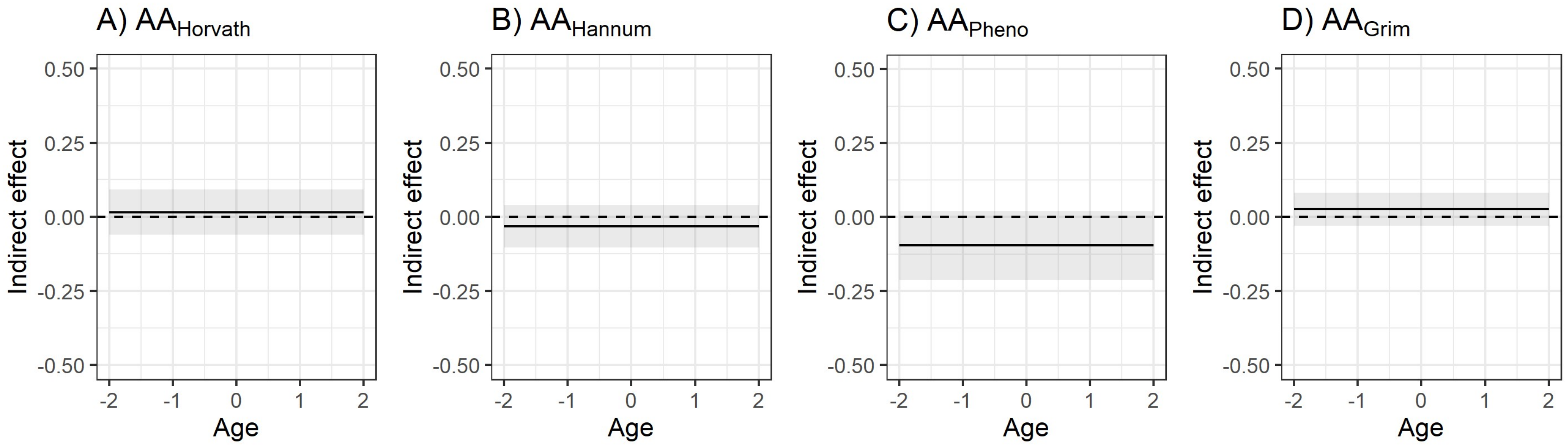

Supplementary Figure S17. Sensitivity analysis: Indirect effects of sex (male) on epigenetic age acceleration (AA) through alcohol use by age (multiple mediator models).

Supplementary Table S5. Multilevel mediation models for opposite-sex twin pairs (151 pairs). Within-twin pair level standardized regression coefficients of the single mediator models are presented.

	**Mediator [a, b]**													
	**Education**		**Body mass index**		**Smoking**		**Alcohol use**		**Sport index**		**Leisure index**		**Work index**	
	**B (SE)**	***p***	**B (SE)**	***p***	**B (SE)**	***p***	**B (SE)**	***p***	**B (SE)**	***p***	**B (SE)**	***p***	**B (SE)**	***p***
**Epigenetic age acceleration regressed on**														
**Mediator** (*b*)														
$AA_{Horvath}$	0.11 (0.09)	.213	**0.24 (0.08)**	**.002**	-0.04 (0.12)	.761	-0.10 (0.08)	.209	**-0.14 (0.06)**	**.024**	0.00 (0.08)	.957	-0.13 (0.07)	.086
AA_{Hannum}	0.06 (0.08)	.472	-0.05 (0.11)	.614	-0.05 (0.09)	.571	-0.12 (0.09)	.179	0.14 (0.08)	.072	0.11 (0.07)	.117	0.01 (0.07)	.895
AA_{Pheno}	0.10 (0.07)	.162	0.07 (0.09)	.437	0.11 (0.11)	.304	-0.07 (0.08)	.376	0.15 (0.09)	.110	0.11 (0.07)	.099	0.04 (0.07)	.598
AA_{Grim}	-0.15 (0.07)	.051	-0.03 (0.11)	.808	**0.36 (0.09)**	**<.001**	0.03 (0.08)	.687	-0.02 (0.09)	.819	-0.09 (0.07)	.167	0.08 (0.06)	.179
**Sex (male)** (*c*)														
$AA_{Horvath}$	**0.12 (0.06)**	**.049**	0.05 (0.06)	.385	0.10 (0.06)	.074	**0.13 (0.06)**	**.033**	0.10 (0.06)	.080	0.10 (0.06)	.095	0.11 (0.06)	.056
AA_{Hannum}	**0.27 (0.05)**	**<.001**	**0.27 (0.06)**	**<.001**	**0.26 (0.06)**	**<.001**	**0.29 (0.06)**	**<.001**	**0.26 (0.05)**	**<.001**	**0.28 (0.05)**	**<.001**	**0.25 (0.05)**	**<.001**
AA_{Pheno}	-0.03 (0.06)	.662	-0.06 (0.06)	.344	-0.05 (0.06)	.375	-0.03 (0.06)	.687	-0.04 (0.06)	.449	-0.02 (0.06)	.754	-0.09 (0.12)	.425
AA_{Grim}	**0.22 (0.05)**	**<.001**	**0.26 (0.06)**	**<.001**	**0.22 (0.06)**	**<.001**	**0.24 (0.06)**	**<.001**	**0.25 (0.05)**	**<.001**	**0.23 (0.05)**	**<.001**	**0.24 (0.05)**	**<.001**
**Mediator regressed on** (*a*)														
Sex (male)	**-0.17 (0.06)**	**.002**	**0.22 (0.06)**	**<.001**	0.07 (0.07)	.286	**0.27 (0.04)**	**<.001**	-0.01 (0.07)	.847	**-0.24 (0.07)**	**.001**	0.07 (0.07)	.332

Notes: B, standardized regression coefficient; SE, standard error; AA, epigenetic age acceleration.
[a] The model was controlled for age as twin pairs participated in measurements at slightly different ages (±1 year).
[b] Significant regression coefficients at the level .05 are presented in bold.

Supplementary Table S6. The association between age and the standardized age-adjusted DNA methylation (DNAm)-based plasma proteins and smoking pack-years modelled as a third to first order polynomial function of age (n = 2240).

	Cubic model [a,b]			Quadratic model [a,b]			Linear model [a,b]		
	B	SE	*p*	B	SE	*p*	B	SE	*p*
Sex (male)									
DNAm ADM	-0.70	0.02	<.001	-0.70	0.02	<.001	-0.70	0.02	<.001
DNAm B2M	-0.01	0.03	.003	-0.01	0.03	.003	-0.10	0.03	.002
DNAm GDF15	-0.17	0.03	<.001	0.00	0.07	.976	.. [d]		
DNAm cystatin C	0.07	0.03	.019	0.07	0.03	.020	.. [d]		
DNAm leptin	-0.94	0.02	<.001	.. [d]			.. [d]		
DNAm PAI-1	0.33	0.03	<.001	.. [d]			.. [d]		
DNAn TIMP-1	0.16	0.03	<.001	0.15	0.03	<.001	0.14	0.03	<.001
DNAm packyrs	0.13	0.03	<.001	0.13	0.03	<.001	0.12	0.03	<.001
Age									
DNAm ADM	-0.30	0.06	<.001	-0.22	0.04	<.001	-0.26	0.02	<.001
DNAm B2M	-0.16	0.09	.067	-0.12	0.06	.042	-0.08	0.03	.009
DNAm GDF15	-0.42	0.10	<.001	-0.30	0.07	<.001	.. [d]		
DNAm cystatin C	-0.25	0.09	.003	-0.25	0.06	<.001	.. [d]		
DNAm leptin	-0.16	0.05	<.001	.. [d]			.. [d]		
DNAm PAI-1	0.29	0.10	.003	.. [d]			.. [d]		
DNAn TIMP-1	-0.21	0.08	.011	-0.13	0.07	.056	-0.05	0.03	.107
DNAm packyrs	-0.03	0.08	.673	-0.04	0.06	.490	0.03	0.03	.290
Age^2									
DNAm ADM	-0.14	0.07	.042	-0.04	0.04	.304	..[d]		
DNAm B2M	0.00	0.09	.989	0.05	0.05	.395			
DNAm GDF15	0.03	0.08	.730	0.33	0.07	<.001			
DNAm cystatin C	0.20	0.09	.031	0.20	0.06	<.001			
DNAm leptin	-0.23	0.05	<.001	.. [d]					
DNAm PAI-1	0.04	0.08	.652	.. [d]					
DNAn TIMP-1	-0.03	0.09	.696	0.08	0.06	.156			
DNAm packyrs	0.09	0.10	.403	0.08	0.06	.203			
Age^3									
DNAm ADM	0.17	0.09	.064	..[e]			..[e]		
DNAm B2M	0.08	0.13	.548						
DNAm GDF15	0.04	0.12	.001						
DNAm cystatin C	0.00	0.12	.985						
DNAm leptin	0.08	0.07	.247						
DNAm PAI-1	-0.34	0.14	.013						
DNAn TIMP-1	0.20	0.12	.094						
DNAm packyrs	-0.02	0.13	.884						
Sex × Age									
DNAm ADM	0.15	0.03	<.001	0.14	0.03	<.001	0.15	0.03	<.001
DNAm B2M	0.12	0.04	.001	0.12	0.04	.001	0.11	0.04	.002
DNAm GDF15	-0.05	0.03	.105	0.03	0.04	.466	.. [d]		

DNAm cystatin C	0.23	0.04	<.001	0.23	0.04	<.001	..[d]		
DNAm leptin	-0.01	0.05	.797	..[d]			..[d]		
DNAm PAI-1	0.17	0.03	<.001	..[d]			..[d]		
DNAn TIMP-1	0.22	0.04	<.001	0.22	0.04	<.001	0.20	0.03	<.001
DNAm packyrs	-0.04	0.04	.313	-0.04	0.04	.323	-0.05	0.04	.167
Sex × Age^2									
DNAm ADM	..[c]			..[c]			..[e]		
DNAm B2M	..[c]			..[c]					
DNAm GDF15	..[c]			-0.16	0.06	.007			
DNAm cystatin C	..[c]			..[c]					
DNAm leptin	..[c]			..[d]					
DNAm PAI-1	..[c]			..[d]					
DNAn TIMP-1	..[c]			-0.06	0.07	.398			
DNAm packyrs				..[c]					
Sex × Age^3									
DNAm ADM	..[c]			..[e]			..[e]		
DNAm B2M	..[c]								
DNAm GDF15	..[c]								
DNAm cystatin C	..[c]								
DNAm leptin	0.09	0.04	.027						
DNAm PAI-1	..[c]								
DNAn TIMP-1	..[c]								
DNAm packyrs	..[c]								

Notes: ADM, adrenomedullin; B2M, beta-2 microglobulin; GDF15, growth differentiation factor 15; PAI-1, plasminogen activation inhibitor 1; TIMP-1, tissue inhibitor metalloproteinase 1; packyrs, pack-years.
B, standardized (STDYX) regression coefficient; SE, standard error.
[a] The model was controlled for zygosity.
[b] SEs were corrected for nested sampling.
[c] The higher order interactions (Sex × Age^2, Sex × Age^3) were freed only when necessary (modification index > 4)
[d] The model was not fitted, because a higher order polynomial model was needed for the measure.
[e] The model did not include the corresponding polynomial term.

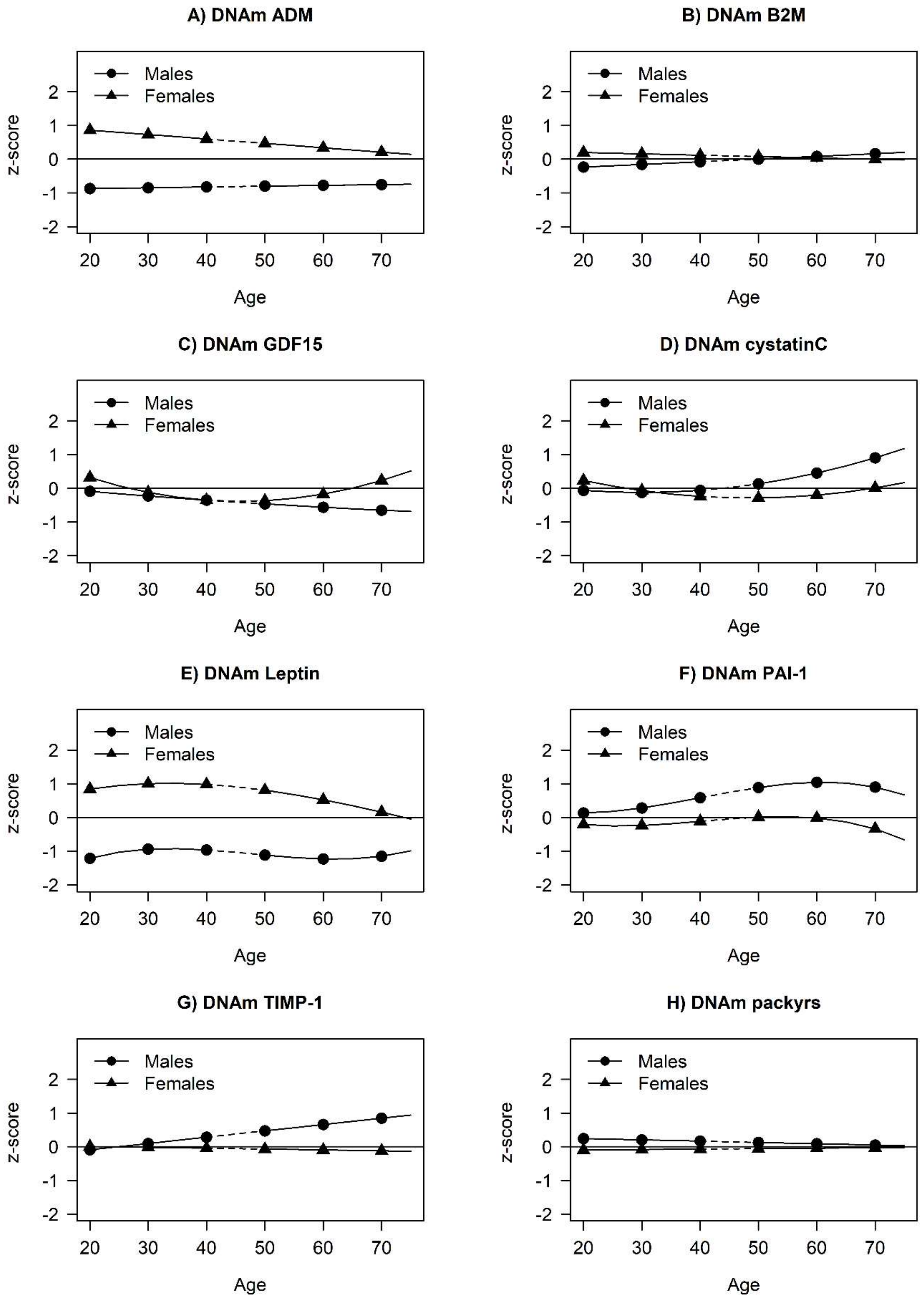

Supplementary Figure S18. DNA methylation (DNAm)-based plasma proteins and smoking pack-years by sex and age in the same-sex twins (n = 2240).
Dotted line segments denote the lack of data for 43- to 49-year-olds. ADM, adrenomedullin; B2M, beta-2 microglobulin; GDF15, growth differentiation factor 15; PAI-1, plasminogen activation inhibitor 1; TIMP-1, tissue inhibitor metalloproteinase 1; packyrs, pack-years.

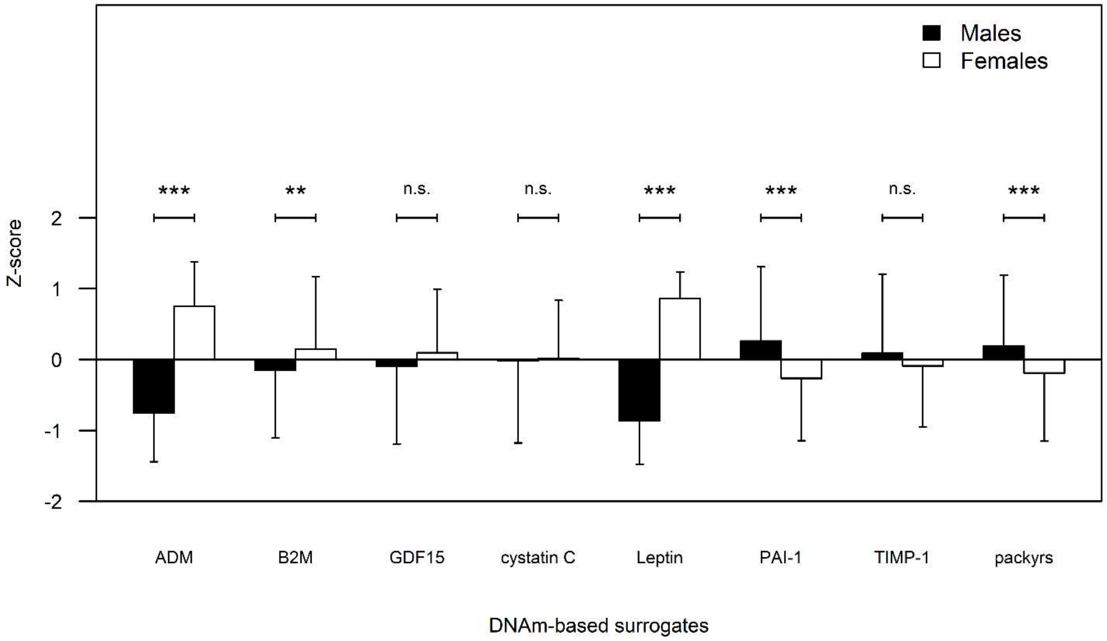

Supplementary Figure S19. The mean levels (and standard deviations) of standardized age-adjusted DNA methylation (DNAm)-based plasma proteins and smoking pack-years by sex in the opposite-sex twin pairs (151 pairs).
ADM, adrenomedullin; B2M, beta-2 microglobulin; GDF15, growth differentiation factor 15; PAI-1, plasminogen activation inhibitor 1; TIMP-1, tissue inhibitor metalloproteinase 1; packyrs, pack-years; ***, $p < .001$; **, $p < .01$; *, $p < .05$, n.s., not significant; p-value for within-twin pair sex difference.
